# Peer-to-Peer Support and Changes in Health and Well-being in Older Adults Over Time

**DOI:** 10.1001/jamanetworkopen.2021.12441

**Published:** 2021-06-15

**Authors:** Rebecca J. Schwei, Scott Hetzel, KyungMann Kim, Jane Mahoney, Kali DeYoung, Jenni Frumer, Ross P. Lanzafame, Jenny Madlof, Alis Simpson, Erika Zambrano-Morales, Elizabeth A. Jacobs

**Affiliations:** 1 BerbeeWalsh Department of Emergency Medicine, University of Wisconsin Madison School of Medicine and Public Health, Madison; 2 Department of Biostatistics and Medical Informatics, University of Wisconsin Madison School of Medicine and Public Health, Madison; 3 Department of Medicine, University of Wisconsin School of Medicine and Public Health, Madison; 4 BioTel Research, Rochester, New York; 5 Next Generation of Holocaust Survivors Inc, Boynton Beach, Florida; 6 Community Place of Greater Rochester, Rochester, New York; 7 Alpert Jewish Family Service of West Palm Beach, West Palm Beach, Florida; 8 Brockport Research Institute, Brockport, New York; 9 California State University, Los Angeles; 10 Department of Medicine and Population Health, University of Texas at Austin Dell Medical School, Austin; 11 now with Maine Medical Center Research Institute, Scarborough

## Abstract

**Importance:**

Literature on peer-to-peer (P2P) programs suggests they improve health and well-being of older adults. Analysis from a previous study showed P2P to be associated with higher rates of hospitalization and no significant differences in rates of emergency department or urgent care visits; however, it is not known whether measures of health and well-being varied by group over time.

**Objective:**

To compare the association between receiving P2P support and secondary outcomes (ie, health status, quality of life, and depressive and anxiety symptoms) with receiving standard community services (SCS) over time.

**Design, Setting, and Participants:**

This cohort study was conducted among a volunteer sample of older adults (≥65 years) who were new to P2P or were already receiving P2P and a corresponding control group. Participants were matched between groups on age, sex, and race/ethnicity. The study was conducted from March 2015 to December 2017 at 3 community-based organizations that delivered P2P in California, Florida, and New York. Data analysis was performed from October 2018 through May 2020.

**Exposures:**

P2P support, provided by trained older adult volunteers.

**Main Outcomes and Measures:**

Mental and physical components of the health status and quality of life measure and depressive and anxiety symptoms were collected over 12 months. The hypothesis was that older adults receiving P2P support would maintain higher health status and quality of life than the SCS group.

**Results:**

A total of 503 participants were screened, 456 participants were enrolled and had baseline data, and 8 participants only had baseline information with no follow-up data, leaving 448 participants (231 [52%] in the SCS group; 217 [48%] in the P2P group; 363 [81%] women; mean [SD] age, 80 [9] years). The P2P group had improvements in mental health (change at 12 months, 1.1 points; 95% CI, −0.8 to 3.0 points) and physical health (change at 12 months, 1.0 points; 95% CI, −0.7 to 2.8 points). However, the difference of differences between the 2 groups did not differ significantly from baseline to 12 months (mental health: 0.2 points; 95% CI −2.3 to 2.7 points; physical health: 1.7 points; 95% CI, −0.6 to 3.9 points). The P2P and SCS groups had a statistically significant difference of differences in anxiety symptoms of 0.36 points (95% CI, 0.04 to 0.61 points). There were no significant differences in depressive symptoms or mental and physical components of the health status and quality of life.

**Conclusions and Relevance:**

These findings suggest that receiving P2P support did not slow the decline of health and well-being in older adults compared with those who received SCS. Baseline imbalance in key characteristics, even after adjusting for the imbalance using the propensity score method, may explain the results. Randomized trials are needed.

## Key Points

**Question:**

Is a community-designed and implemented peer-to-peer (P2P) support program associated with changes in measures of health and well-being, such as depressive or anxiety symptoms, vs standard community services (SCS)?

**Findings:**

In this cohort study of 448 older adults, the P2P group had a significant increase in anxiety symptoms compared with the SCS group. The P2P group had improvements in mental and physical health over the year, but these did not differ significantly from the SCS group.

**Meaning:**

In this study, the results were mixed and did not show that receiving P2P support was associated with improvements in or a slower decline of health and well-being compared with the SCS group.

## Teaser

This cohort study compares health status, quality of life, and depressive and anxiety symptoms between older adults receiving peer-to-peer support vs those receiving standard community services.

## Introduction

The US Census Bureau projects that in 2030 people 65 years and older will make up more than 20% of the total population in the US.^1^ Overall, 76% of adults aged 65 years and older report their health as good, very good, or excellent. Despite this, functional limitations are common.^2^ In 2013, approximately 44% of people aged 65 years and older enrolled in Medicare reported a functional limitation.^3^

Peer support programs have been implemented in a wide variety of health care and community settings for a variety of reasons ranging from diabetes management to support with mental health conditions to improvements in birth outcomes. At least 35 scholarly reviews have examined the benefits of peer support programs, and approximately 65% identified benefits.^4^ Broadly, peer support programs have been associated with improvements in clinical and quality-of-life outcomes^4,5,6^ and reductions in hospitalizations.^7,8,9^ Additionally, these programs are feasible in a wide range of settings^10,11^ and are cost-effective.^7,12,13,14^

Peer support programs have been suggested for low- and middle-income countries as a way to improve social and physical well-being in isolated older adults.^15^ Previous studies have found these programs support older adults’ health and well-being^16,17,18,19,20^ and can promote independence.^20^ In peer-to-peer (P2P) support programs, 1 older adult is matched with a less-able older adult in the same community as a way to support that less-able older adult. Peer supporters provide a variety of types of assistance, including visiting clients in their homes, taking them to the grocery store or a doctor’s appointment, and providing a regular social connection. Several studies have investigated the association of P2P support with older adults’ general health and well-being.^16,17,18,19,20^ One 1999 program evaluation with a sample size of 22 found older adults who were experiencing loneliness and were randomized to receive a weekly visitor from a trained undergraduate student had improved life satisfaction and improvements in worth and social integration after 6 weeks of follow-up compared with the group of older adults who were randomized to receive no visitor.^16^ These findings have been replicated in studies that found improvements in physical health, general health, social function, and satisfaction among socially isolated older adults.^18,19,20^ Likewise, decreases in anxiety and depressive symptoms^17,19,21^ and increased socialization^17,19^ have been reported in older adults who receive P2P support. One qualitative study published in 2012 suggested that volunteer programs that addressed recipients’ social, emotional, and mobility needs can contribute to older adults and adults with disabilities remaining in their homes.^20^

While there is substantial literature suggesting that receiving P2P support improves general health and well-being for older adults, many of these studies were qualitative,^16,17,20,21^ had small sample sizes,^18,21^ or did not have a control group for comparison.^19^ We sought to expand on the existing literature by comparing the differences in a variety of measures of health and well-being over 1 year between older adults receiving P2P support and older adults who were receiving standard community services (SCS) using a large sample size across 3 different organizations with a 12-month follow-up period. We have previously published^22^ findings from this study that compared hospital, emergency department, and urgent care utilization between the P2P group and the SCS group. There was a statistically significant higher rate of hospitalization in the P2P group than the SCS group. There were no significant differences in the rates of emergency department or urgent care visits or in the composite outcome of any health care utilization between the 2 groups. The secondary objective of the study was to compare the association between receiving P2P support and health status, quality of life, and depressive and anxiety symptoms compared with receiving SCS. Given the improvements in general health and depression and anxiety symptoms found in previous studies examining P2P programs,^17,18,19,20,21^ we hypothesized that older adults receiving P2P support would maintain better health status and quality of life and have fewer depressive and anxiety symptoms than the SCS group.

## Methods

The first goal of this study was to compare the association of P2P community support in preventing known risk factors for moving out of the community: frequent health care utilization measured by hospitalization, urgent care, and emergency department use in an at-risk older adult population relative to a SCS group.^22^ As a secondary aim, we compared the association of receiving P2P support on measures of health and well-being vs receiving SCS. The results of the second aim are presented here. We conducted an longitudinal cohort study, and the P2P community support programs that participated in this project were located in Los Angeles, California; Rochester, New York; and West Palm Beach, Florida. All study activities were approved by the Western Institutional Review Board, and the trial was registered on ClinicalTrials.gov (NCT02308696). A full protocol is included in eAppendix 1 in the Supplement. All participants provided written informed consent. This manuscript follows the Strengthening the Reporting of Observational Studies in Epidemiology (STROBE) reporting guideline for observational studies.

### Study Population

We recruited older adults in the P2P group and the SCS group and frequency matched participants on age (<70, 70-79, and ≥80 years), sex (male vs female), and race/ethnicity (African American/Black, Asian, White, Hispanic, and other) during the recruitment phase of the study. Race/ethnicity were self-reported based on investigator-designated categories; individuals who did not select 1 of the predefined categories were included in the other group. We collected race/ethnicity data to ensure we were capturing a broad range of participants in the study. At the beginning of the study, we looked at the sex, race/ethnicity, and age breakdown of participants in the P2P group at each site and developed recruitment buckets that we tried to fill. Enrollment goals at each site were set based on the size of the P2P program and an overall power calculation for the primary outcome of interest (ie, health care utilization). Enrollment buckets ensured we were recruiting in a balanced way so that the P2P and SCS were frequency matched on age, sex, and race/ethnicity categories by the end of recruitment.

To be eligible, adults had to be 65 years or older, live independently in their community year-round, and meet the community-defined criteria for receiving P2P support. Older adults met community-defined criteria to receive P2P support if they were living at or below poverty level or on a fixed income that did not meet their living expenses; were socially isolated; had chronic illnesses; or frequently used community services or resources that the organization offered (such as social gatherings, a meal, or bereavement services). Participants were excluded if they lived in assisted living or a nursing home. We excluded adults who had cognitive impairment, defined as having a score of 25 or lower on the 11-question Telephone Interview for Cognitive Status instrument, as they could not provide informed consent.^23^ To be included in the P2P group, older adults had to be receiving P2P support. Older adults in Los Angeles were new to the P2P program, and those in Rochester and West Palm Beach had been previously enrolled in the P2P program. To be in the SCS group, older adults had to have met the qualifications for receiving P2P support from the community organization as determined by the research coordinator at each site but not be engaged in the P2P program.

We recruited participants in 2014 and 2015 from 3 sites: Alpert Jewish Family Service of Palm Beach County; Jewish Family Service of Los Angeles; and The Community Place of Greater Rochester. At each site we recruited from existing and newly created P2P support programs. For the control group, we recruited from other programs for older adults that were run either by the site (eg, senior center activities, such as community meals, foster grandparent programs, bereavement programs), by a different organization (eg, Meals on Wheels, ≥55-years communities), and from community events. Older adults did not need to be receiving any services to be enrolled in the control group; however, many received some services as a result of our recruitment methods. The study coordinator contacted interested participants. If the older adult passed the eligibility screening, we scheduled an initial study visit that took 45 to 90 minutes. During the visit, we completed informed consent and the baseline survey. We followed up participants for 1 year and collected additional information via a combination of telephone and in-person visits at 3, 6, 9, and 12 months. We collected information on the measures of health and well-being at baseline, 6 months, and 12 months only. We contacted participants at 3 months and 9 months to keep them engaged in the study and to assess the primary outcomes of health care utilization. If a participant let us know that they had moved to a higher level of care (eg, home to independent care) during a follow-up visit, we finished data collection for that time point and then they were excluded from future data collection, as they had met the main outcome of the study. If a participant was unable to answer the survey questions, we skipped the question; proxy responses were not allowed. We entered survey responses directly into DatStat (DatStat), a cloud-based data collection and management system. Participants received a $25 gift card for completing the baseline survey and a $75 gift card for completing final data collection.

### Intervention Group: P2P Services

All 3 study sites had P2P programs with standardized core elements as outlined in Table 1, including a similar definition of who qualifies for P2P support. Peers had to be 55 years or older, and most received small stipends for volunteering. Older adults in the P2P support programs continued to have access to standard services even while they were enrolled in the P2P group.

**Table 1.** Core Elements of the Peer-to-Peer Community Support Intervention

Element	Description
Goal	To promote successful aging in place among frail older adults
Duration of peer-to-peer relationship	From enrollment until patient transitioned to more advanced care, left the area, or died
Target population	Older adults at risk of a decline in health or placement in long-term care
Referral process	Self-referral or referral by case managers from the community organization or local health care organizations
Volunteer selection	Adults ≥55 y who were able to dedicate 20 h/wk to peer support with a minimum 1-y commitment
Volunteer training	
Initial training	0-20 h
Training modules	Developing a peer-to-peer support relationship Importance of companionship Basic health and emotional health needs of at-risk older adults How to provide emotional support Overview of services provided by the organization and by the community and how to access them Trouble-shooting particular issues that might arise in a relationship
Monthly in-service trainings	1-2 h Relevant topic
Expectations of volunteers	Attend all trainings Attend ≥60% of monthly in-service trainings Provide ≥20 h of peer support per month Contact assigned peers on a regular basis
Peer client load per volunteer	Minimum of 2 to a maximum of 10 peer clients
Shaped to meet the local community needs	Each program added training on particular issues or community resources unique to the community they serve

### Control Group: SCS

Older adults in the SCS group had access to all the services that each participating community organization provided, with the exception of P2P. The standard aging services offered at each site included health, wellness, socialization, and enrichment activities, case management and counseling, resource referrals, food pantry, and meal delivery.

### Outcomes

We measured health and well-being outcomes at baseline, 6 months, and 12 months. All measures were translated to Spanish and have been shown to be valid or have high reliability in Spanish.^24,25,26,27,28,29,30,31,32,33^ We measured health status and quality of life (12-item Short Form Survey; range, 0-100; higher scores indicate better health and quality of life),^34,35,36,37^ depressive symptoms (Center for Epidemiological Studies Depression; range, 0-10; higher scores indicate more symptoms),^38^ anxiety symptoms (Geriatric Anxiety Inventory; range, 0-5; higher scores indicate more symptoms),^39^ loneliness (Short Scale for Measuring Loneliness in Large Surveys; range, 0-3; higher score indicates greater loneliness),^40^ self-efficacy (General Self-Efficacy Scale; range, 1-4; higher scores indicate higher self-efficacy),^41^ resilience (Brief Resilience Scale; range, 1-5; higher scores indicate greater resilience),^42^ social support (Social Support Survey; range, 1-5; higher scores indicate less social support),^43^ activities of daily living (ADLs; range, 0-6; higher scores indicate greater dependence),^44,45^ instrumental ADLs (IADLs; range, 0-8; higher scores indicate greater dependence),^46^ mobility and strength (Rosow-Bresleau; range, 0-3; higher scores indicate less mobility and strength),^47,48^ and physical function (Nagi; range, 1-5; higher scores indicate less function).^26,48,49,50^ The outcomes included in this article are a subset of the questions we asked patients. We report here the outcome measures related to health and well-being. Further descriptions of the outcomes are included in eTable 1 in the Supplement.

### Sociodemographic Characteristics

We collected data on age, race and ethnicity (African American/Black, Asian, White, Hispanic, or other), marital status (never married, married, widowed, or separated or divorced), current living arrangements (alone, with partner, with other family member, and/or other caregiver), household income, and recorded educational attainment in terms of years of formal schooling completed. We also documented which language the surveys were completed in (English or Spanish).

### Statistical Analysis

We compared baseline health status and quality of life, depressive symptoms, anxiety symptoms, loneliness, self-efficacy, resilience, social support, ADLs, IADLs, basic mobility and strength, and physical function between the 2 groups using 2-sample tests at a 2-tailed significance level of .05 without adjustment for multiplicity of outcomes and testing because these analyses are of secondary outcomes and are exploratory. The baseline characteristics of the P2P and SCS groups were not similar except for age, sex, and race/ethnicity, so we adjusted for these differences using a propensity score for each participant.^51,52^ A full description of how the propensity score was built is included in our previous publication.^22^ Briefly, we conducted a logistic regression analysis using intervention group status (P2P vs SCS) as the outcome and baseline characteristics as covariates to understand the association between group status and baseline measures of previous health care utilization, health status, well-being, and demographic information. We included baseline participant characteristics that were associated with being in the P2P group at the *P* < .15 level to estimate a propensity score for each participant. We weighted our final regression models using the inverse propensity score for each participant.^22^

Using a mixed-effects longitudinal data analysis model with group (2-level factor), time (3-level factor), and their interaction as fixed effects, site as a covariate, participant as a random effect, and inverse propensity score as a weight, we estimated mean (95% CI) change from baseline in each measure at 6 months and 12 months. Using this model, we were able to test for differences between groups overall and by site and change from baseline for the health and well-being variables while accounting for systematic differences in baseline characteristics between groups. We had missing data due to random drop-out of participants and because participants died or transitioned to a higher level of care before month 12. All analyses were conducted using R version 3.5 (R Project for Statistical Computing). A full statistical analysis plan is included as eAppendix 2 in the Supplement.

## Results

The Figure presents the flowchart describing participant enrollment by study group. A total of 503 participants were screened, and the final sample included 456 adults (234 [51%] in the SCS group; 222 [49%] in the P2P group). The 2 groups were balanced within each site by age, sex, and race/ethnicity (factors used for frequency-matching between the P2P and the SCS groups). Table 2 describes the demographic characteristics by study group and site using the raw data collected directly from participants.

**Figure.**
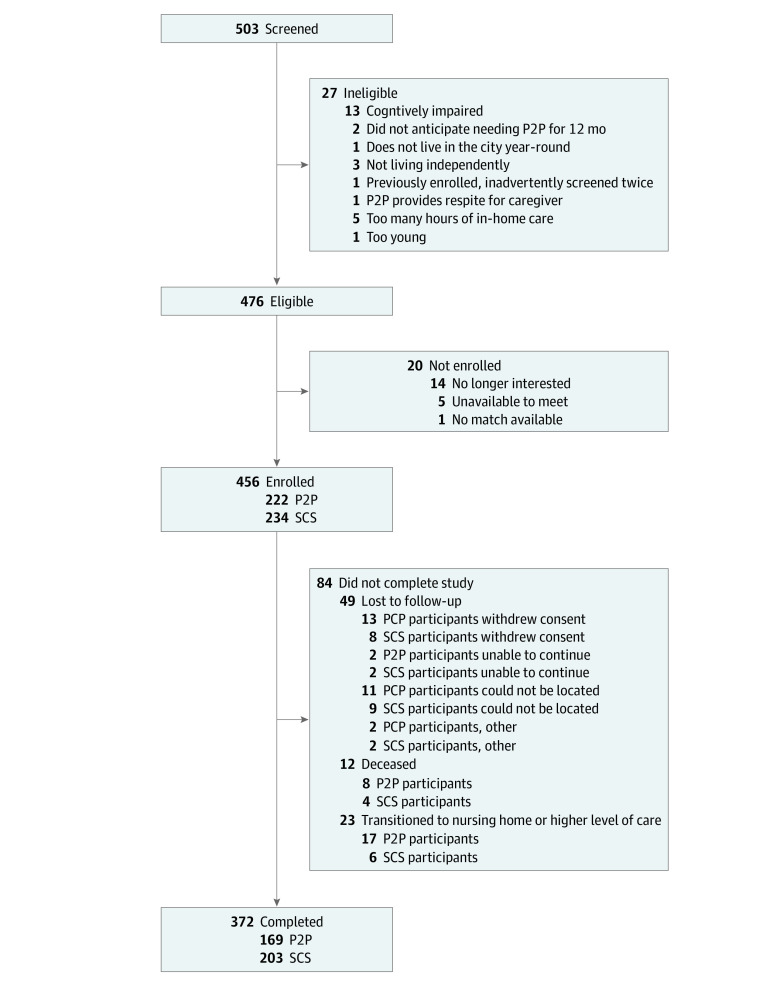
Study Flowchart P2P indicates peer-to-peer; SCS, standard community services.

**Table 2.** Demographic Characteristics by Study Group and Site

Characteristic	Overall			Los Angeles			Rochester			West Palm Beach		
	Participants, No. (%)		*P* value	Participants, No. (%)		*P* value	Participants, No. (%)		*P* value	Participants, No. (%)		*P* value
	SCS (n = 234)	P2P (n = 222)		SCS (n = 40)	P2P (n = 35)		SCS (n = 103)	P2P (n = 100)		SCS (n = 91)	P2P (n = 87)	
Sex												
Female	185 (79.1)	185 (83.3)	.30	27 (67.5)	25 (71.4)	.91	87 (84.5)	91 (91.0)	.23	71 (78.0)	68 (79.3)	.98
Male	49 (20.9)	37 (16.7)		13 (32.5)	10 (28.6)		16 (15.5)	9 (9.0)		20 (22.0)	19 (20.7)	
Race/ethnicity												
African American	25 (10.7)	24 (10.8)	.81	1 (2.5)	1 (2.9)	.61	24 (23.3)	23 (23.0)	.71	0 (0.0)	0 (0.0)	>.99
Asian	2 (0.9)	2 (0.9)		1 (2.5)	2 (5.7)		1 (1.0)	0 (0.0)		0 (0.0)	0 (0.0)	
Hispanic	24 (10.3)	21 (9.5)		6 (15.0)	2 (5.7)		17 (16.5)	18 (18.0)		1 (1.1)	1 (1.1)	
White	183 (78.2)	173 (77.9)		32 (80.0)	30 (85.7)		61 (59.2)	57 (57.0)		90 (98.9)	86 (98.9)	
Other^a^	0 (0.0)	2 (0.9)		0 (0.0)	0 (0.0)		0 (0.0)	2 (2.0)		0 (0.0)	0 (0.0)	
Age, mean (SD)	79.1 (8.7)	80.4 (9.3)	.13	77.4 (8.3)	81.0 (8.9)	.71	76.0 (8.1)	76.8 (8.6)	.53	83.3 (7.9)	84.3 (8.6)	.44
Marital status												
Never married	18 (7.9)	24 (10.9)	.004	8 (20.5)	4 (11.4)	.09	8 (8.1)	13 (13.1)	.56	2 (2.2)	7 (8.0)	.005
Married	51 (22.3)	22 (10.0)		7 (17.9)	1 (2.9)		17 (17.2)	12 (12.1)		27 (29.7)	9 (10.3)	
Widowed	100 (43.7)	112 (50.7)		12 (30.8)	17 (48.6)		42 (42.4)	40 (40.4)		46 (50.5)	55 (63.2)	
Divorced or separated	60 (26.2)	63 (28.5)		12 (30.8)	13 (37.1)		32 (32.3)	34 (34.3)		16 (17.6)	16 (18.4)	
Lives alone	162 (69.8)	177 (79.7)	.02	30 (76.9)	25 (71.4)	.78	70 (68.6)	77 (77.0)	.24	62 (68.1)	75 (86.2)	.007
Household income, $												
<10 000	20 (11.7)	35 (21.3)	<.001	7 (21.2)	5 (16.7)	.11	12 (13.0)	22 (24.2)	.02	1 (2.2)	8 (18.6)	.008
10 000-14 999	50 (29.2)	61 (37.2)		10 (30.3)	18 (60.0)		32 (34.8)	34 (37.4)		8 (17.4)	9 (20.9)	
15 000-24 999	47 (27.5)	48 (29.3)		12 (36.4)	6 (20.0)		24 (26.1)	26 (28.6)		11 (23.9)	16 (37.2)	
25 000-49 999	44 (25.7)	15 (9.1)		4 (12.1)	1 (3.3)		21 (22.8)	6 (6.6)		19 (41.3)	8 (18.6)	
≥50 000	10 (5.8)	5 (3.0)		0 (0.0)	0 (0.0)		3 (3.3)	3 (3.3)		7 (15.2)	2 (4.7)	
Schooling, mean (SD), y	13.5 (3.2)	13.1 (3.5)	.21	13.4 (4.1)	13.6 (3.3)	.76	13.3 (3.5)	12.7 (3.8)	.27	13.8 (2.2)	13.3 (3.3)	.27
Spanish-language preference	8 (3.9)	13 (6.6)	.46	3 (7.5)	1 (2.9)	.71	5 (5.2)	12 (14.3)	.07	0	0	NA

Abbreviations: P2P, peer-to-peer; SCS, standard community services.

^a^ Individuals who did not identify as any of the other categories were included in the other group.

There were 75 participants (16%) from Los Angeles, 203 (46%) from Rochester, and 178 (39%) from West Palm Beach. Overall, 370 (81%) were female; 356 (78%) self-identified as White; 49 (11%) as African American/Black; and 45 (10%) as Hispanic. Participants in our study had a mean (SD) age of 80 (9) years. In the SCS group, 162 participants (70%) lived alone, and in the P2P group, 177 (80%) lived alone; this difference was statistically significant (*P* = .02). The SCS group had significantly greater household income compared with the P2P group (eg, 10 [6%] earning ≥$50 000 vs 5 [3%]; *P* < .001). A total of 21 participants (5%) preferred to conduct the study in Spanish. A total of 8 participants only had baseline information with no follow-up data, leaving 448 participants (231 [52%] in the SCS group; 217 [48%] in the P2P group; 363 [81%] women; mean [SD] age, 80 [9] years).

### Measures of Self-reported Disability

Using the raw data, at baseline, the SCS group had a significantly higher mean (SD) score on the physical health component of the health status and quality of life than the P2P group (41.2 [12.7] vs 36.0 [13.1]; *P* < .001), and this was true at both Los Angeles and West Palm Beach (Table 3). Additionally, the P2P group self-reported more median (range) limitations with IADLs than the SCS group (1.0 [0.0-3.0] vs 0.0 [0.0-1.0]; *P* < .001), more issues with basic mobility and strength (mean [SD] score, 1.9 [1.0] vs 1.3 [1.0]; *P* < .001), and more challenges with physical function (mean [SD] score, 3.0 [1.4] vs 2.3 [1.5]; *P* < .001). In the propensity score model, the P2P group had improvements in physical health (baseline, 35.3 points [95% CI, 3.5 to 37.2 points]; 12 months, 36.4 points [95% CI, 4.4 to 38.3 points]; change from baseline, 1.0 points [95% CI, −0.7 to 2.8 points]). However, the DoD between the 2 groups did not differ significantly from baseline to 12 months (1.7 points; 95% CI, −0.6 to 3.9 points). None of the other measures of self-reported disability were significantly different from baseline to 6 or 12 months between P2P and SCS groups (Table 4). In Los Angeles, the SCS group had a decrease in physical health, and the P2P group had an increase, which resulted in a significant difference in differences (DoD) from baseline to 12 months of 5.9 points (95% CI, 0.7 to 11.1 points) (eTable 2 in the Supplement). In Los Angeles, the SCS reported a 0.26-point increase in challenges with mobility and strength from baseline to 12 months, and the P2P group reported a 0.17-point improvement, for a DoD of −0.43 (95% CI, −0.80 to −0.60) points. There were no differences in self-reported measures of disability from baseline to 6 or 12 months at Rochester and West Palm Beach.

**Table 3.** Measures of Health and Well-being at Baseline by Study Group and by Site

Measure	Overall			Los Angeles			Rochester			West Palm Beach		
	Score, mean (SD)		*P* value	Score, mean (SD)		*P* value	Score, mean (SD)		*P* value	Score, mean (SD)		*P* value
	SCS (n = 234)	P2P (n = 222)		SCS (n = 40)	P2P (n = 35)		SCS (n = 103)	P2P (n = 100)		SCS (n = 91)	P2P (n = 87)	
Health status and quality of life												
Mental health	52.3 (12.7)	49.3 (13.4)	.02	46.5 (14.0)	49.8 (14.2)	.33	54.4 (12.0)	51.4 (11.7)	.08	52.3 (12.2)	46.7 (14.6)	.007
Physical health	41.2 (12.7)	36.0 (13.1)	<.001	39.9 (13.7)	31.1 (11.9)	.004	39.1 (12.6)	38.3 (13.3)	.66	44.0 (11.9)	35.3 (12.8)	<.001
Symptoms^a^												
Depressive	3.6 (1.8)	3.9 (1.9)	.05	3.8 (2.0)	3.9 (1.5)	.88	3.6 (1.7)	3.9 (1.8)	.13	3.5 (1.7)	3.9 (2.0)	.18
Anxiety	1.4 (1.7)	1.8 (1.8)	.02	1.9 (1.8)	1.9 (1.9)	.92	1.4 (1.6)	1.6 (1.7)	.33	1.2 (1.6)	1.9 (1.9)	.007^a^
Loneliness^a^	1.5 (0.6)	1.7 (0.7)	<.001	1.8 (0.7)	1.8 (0.7)	.87	1.6 (0.6)	1.6 (0.6)	.88	1.3 (0.5)	1.8 (0.7)	<.001
Self-efficacy	3.4 (0.5)	3.2 (0.6)	<.001	3.4 (0.5)	3.2 (0.6)	.17	3.4 (0.5)	3.3 (0.6)	.14	3.5 (0.6)	3.2 (0.7)	<.001
Resilience	3.6 (0.9)	3.4 (0.8)	.07	3.2 (1.1)	3.4 (1.0)	.30	3.6 (0.7)	3.5 (0.8)	.30	3.7 (0.8)	3.4 (0.8)	.01
Social support^a^	3.7 (1.2)	3.6 (1.0)	.20	2.9 (1.3)	3.5 (1.0)	.03	3.8 (1.1)	3.6 (1.0)	.16	3.9 (1.1)	3.6 (1.0)	.03
Self-reported disability^a^												
ADLs, median (range)	0.0 (0.0-0.0)	0.0 (0.0-1.0)	<.001	0.0 (0.0-0.0)	0.0 (0.0-1.0)	.05	0.0 (0.0-0.0)	0.0 (0.0-1.0)	.11	0.0 (0.0-0.0)	0.0 (0.0-1.0)	.02
Instrumental ADLs, median (range)	0.0 (0.0-1.0)	1.0 (0.0-3.0)	<.001	0.0 (0.0-1.0)	2.0 (1.0-3.0)	<.001	1.0 (0.0-2.0)	1.0 (1.0-2.0)	.005	0.0 (0.0-0.0)	1.0 (0.0-2.0)	<.001
Mobility/strength	1.3 (1.0)	1.9 (1.0)	<.001	1.2 (0.9)	2.2 (0.8)	<.001	1.4 (1.1)	1.6 (1.0)	.20	1.3 (1.0)	2.0 (1.0)	<.001
Physical function	2.3 (1.5)	3.0 (1.4)	<.001	2.5 (1.7)	3.4 (1.0)	.07	2.6 (1.4)	3.0 (1.5)	.06	1.9 (1.4)	3.1 (1.4)	<.001

Abbreviations: ADLs, activities of daily living; P2P, peer-to-peer; SCS, standard community services.

^a^ Smaller numbers indicate better health.

**Table 4.** Health and Wellness Measures Over Time and Difference of Differences at 6 and 12 Months Between P2P and SCS, Inverse Probability Weighted Model With 448 Participants

Measure	Score (95% CI)			6 mo		12 mo	
	Baseline	6 mo	12 mo	Change from baseline (95% CI)	Difference of differences (95% CI)	Change from baseline (95% CI)	Difference of differences (95% CI)
Health status and quality of life							
Mental health component							
SCS	50.4 (48.6 to 52.2)	50.7 (48.9 to 52.5)	51.3 (49.4 to 53.1)	0.3 (−1.3 to 1.9)	1.8 (−0.7 to 4.3)	0.9 (−0.7 to 2.5)	0.2 (−2.3 to 2.7)
P2P	48.9 (47.0 to 50.7)	50.9 (48.9 to 53.0)	49.9 (47.9 to 52.0)	2.1 (0.2 to 4.0)		1.1 (−0.8 to 3.0)	
Physical health component							
SCS	41.2 (39.5 to 43.0)	40.4 (38.6 to 42.4)	40.6 (38.8 to 42.4)	−0.9 (−2.3 to 0.5)	1.0 (−1.2 to 3.3)	−0.6 (−2.1 to 0.8)	1.7 (−0.6 to 3.9)
P2P	35.3 (33.5 to 37.2)	35.5 (33.5 to 37.5)	36.4 (34.4 to 38.3)	0.2 (−1.6 to 1.9)		1.0 (−0.7 to 2.8)	
Depressive symptoms^a^							
SCS	3.77 (3.53 to 4.02)	3.21 (2.96 to 3.45)	3.38 (3.12 to 3.64)	−0.57 (−0.80 to −0.33)	0.24 (−0.14 to 0.62)	−0.39 (−0.65 to −0.14)	0.04 (−0.35 to 0.44)
P2P	3.86 (3.60 to 4.13)	3.54 (3.26 to 3.81)	3.51 (3.23 to 3.80)	−0.33 (−0.62 to −0.03)		−0.35 (−0.65 to −0.05)	
Anxiety symptoms^a^							
SCS	1.61 (1.38 to 1.84)	1.31 (1.08 to 1.55)	1.31 (1.07 to 1.55)	−0.30 (−0.47 to −0.12)	0.01 (−0.26 to 0.29)	−0.30 (−0.48 to −0.12)	0.32 (0.04 to 0.61)^b^
P2P	1.82 (1.57 to 2.06)	1.54 (1.28 to 1.79)	1.84 (1.58 to 2.10)	−0.28 (−0.50 to −0.07)		0.02 (−0.20 to 0.24)	
Loneliness^a^							
SCS	1.56 (1.47 to 1.65)	1.52 (1.43 to 1.61)	1.55 (1.46 to 1.64)	−0.04 (−0.11 to 0.03)	0.00 (−0.11 to 0.10)	−0.01 (−0.08 to 0.06)	0.06 (−0.05 to 0.16)
P2P	1.72 (1.63 to 1.81)	1.68 (1.58 to 1.77)	1.77 (1.67 to 1.86)	−0.04 (−0.12 to 0.04)		0.05 (−0.03 to 0.13)	
Self-efficacy							
SCS	3.43 (3.35 to 3.51)	3.35 (3.27 to 3.43)	3.29 (3.21 to 3.37)	−0.08 (−0.15 to −0.02)	0.05 (−0.05 to 0.15)	−0.14 (−0.21 to −0.08)	0.07 (−0.03 to 0.17)
P2P	3.26 (3.18 to 3.35)	3.23 (3.15 to 3.32)	3.19 (3.10 to 3.28)	−0.03 (−0.11 to 0.05)		−0.07 (−0.15 to 0.01)	
Resilience							
SCS	3.58 (3.47 to 3.69)	3.46 (3.35 to 3.58)	3.63 (3.52 to 3.75)	−0.12 (−0.22 to −0.02)	0.11 (−0.04 to 0.26)	0.05 (−0.05 to 0.15)	−0.23 (−0.38 to −0.08)^b^
P2P	3.46 (3.34 to 3.57)	3.45 (3.32 to 3.57)	3.28 (3.15 to 3.40)	−0.01 (−0.13 to 0.11)		−0.18 (−0.30 to −0.06)	
Social support^a^							
SCS	3.62 (3.47 to 3.76)	3.55 (3.40 to 3.70)	3.72 (3.57 to 3.87)	−0.07 (−0.17 to 0.04)	0.08 (−0.10 to 0.25)	0.10 (0.00 to 0.21)	−0.04 (−0.21 to 0.12)
P2P	3.47 (3.32 to 3.62)	3.48 (3.32 to 3.4)	3.53 (3.37 to 3.69)	0.01 (−0.12 to 0.14)		0.06 (−0.07 to 0.19)	
Self-reported disability^a^							
Activities of daily living							
SCS	0.28 (0.17 to 0.39(	0.28 (0.17 to 0.39)	0.35 (0.24 to 0.46)	0.00 (−0.09 to 0.09)	0.04 (−0.10 to 0.19)	0.07 (−0.02 to 0.17)	−0.05 (−0.20 to 0.09)
P2P	0.47 (0.35 to 0.58)	0.51 (0.39 to 0.63)	0.48 (0.36 to 0.61)	0.04 (−0.07 to 0.15)		0.02 (−0.10 to 0.13)	
Instrumental activities of daily living							
SCS	0.93 (0.68 to 1.19)	1.29 (1.03 to 1.55)	1.10 (0.83 to 1.37)	0.36 (0.13 to 0.59)	0.04 (−0.33 to 0.40)	0.17 (−0.08 to 0.41)	0.00 (−0.38 to 0.38)
P2P	1.70 (1.43 to 1.97)	2.02 (1.73 to 2.31)	1.87 (1.68 to 2.16)	0.32 (0.04 to 0.61)		0.17 (−0.12 to 0.46)	
Basic mobility and strength							
SCS	1.49 (1.35 to 1.62)	1.24 (1.10 to 1.38)	1.49 (1.34 to 1.69)	−0.24 (−0.35 to −0.13)	0.08 (−0.10 to 0.25)	0.00 (−0.12 to 0.12)	0.05 (−0.14 to 0.23)
P2P	1.87 (1.73 to 2.02)	1.71 (1.55 to 1.86)	1.92 (1.76 to 2.07)	−0.17 (−0.30 to −0.03)		0.05 (−0.09 to 0.19)	
Physical function							
SCS	2.43 (2.22 to 2.63)	2.09 (1.88 to 2.29)	2.27 (2.06 to 2.48)	−0.34 (−0.51 to −0.17)	0.09 (−0.18 to 0.37)	−0.15 (−0.34 to 0.03)	0.10 (−0.18 to 0.39)
P2P	3.03 (2.81 to 3.24)	2.78 (2.55 to 3.01)	2.98 (2.75 to 3.21)	−0.25 (−0.46 to −0.03)		−0.05 (−0.27 to 0.17)	

Abbreviations: P2P, peer-to-peer; SCS, standard community services.

^a^ Smaller numbers indicate better health.

^b^ *P* < .05.

### Measures of Emotional Health

Using the raw data, at baseline the SCS group had significantly better mental health that the P2P group (mean [SD] score, 52.3 [12.7] vs 49.3 [13.4]; *P* = .02), had significantly fewer anxiety symptoms (mean [SD] symptoms, 1.4 [1.7] vs 1.8 [1.8]; *P* = .02), were significantly less lonely (mean [SD] score, 1.5 [0.6] vs 1.7 [0.7]; *P* < .001), and had higher mean (SD) self-efficacy scores (3.4 [0.5] vs 3.2 [0.6]; *P* <  .001). These baseline differences also existed at West Palm Beach.

In the propensity score model, the P2P group had improvements in mental health (baseline, 48.9 points [95% CI, 47.0 to 50.7 points]; 12 months, 49.9 points [95% CI, 47.9 to 52.0 points]; change from baseline, 1.2 points [95% CI, −0.8 to 3.0 points]). The SCS group also had improvements in mental health (baseline, 50.4 points [95% CI, 48.6 to 52.2 points]; 12 months, 51.3 points [95% CI, 49.4 to 53.1 points]; change from baseline, 0.9 points [95% CI, −0.7 to 2.5 points]). The DoD between the 2 groups did not differ significantly from baseline to 12 months (0.2 points; 95% CI −2.3 to 2.7 points). The P2P group had a 0.02-point increase in anxiety symptoms, whereas the SCS group had a 0.30-point decrease in anxiety symptoms from baseline to 12 months, for a DoD of 0.32 points (95% CI, 0.04 to 0.61) points. For resilience, the P2P group had a 0.18-point decrease and the SCS group had a 0.05-point increase from baseline to 12 months, for a DoD of −0.23 (95% CI, −0.38 to −0.08). Both P2P and SCS groups experienced a reduction in self-efficacy and depressive symptoms from baseline to 12 months, but there were no significant differences between the two groups (self-efficacy: SCS, −0.14 pints [95% CI, −0.21 to −0.08 points]; P2P, −0.07 points [95% CI, −0.15 to 0.01 points]; DoD, 0.07 points [95% CI, −0.03 to 0.17 points]; depressive symptoms: SCS, −0.39 points [95% CI, −0.65 to −0.14 points]; P2P, −0.35 points [−0.65 to −0.05 points]; DoD: 0.04 points [95% CI, −0.35 to 0.44 points]).

At West Palm Beach, the SCS group had a 0.47-point decrease in anxiety symptoms, and the P2P group had a 0.28-point increase in anxiety symptoms from baseline to 12 months, which resulted in a DoD of 0.75 points (95% CI, 0.36 to 1.15) (eTable 3 in the Supplement). The P2P group at West Palm Beach had a decrease in resilience, while the SCS had an increase in resilience from baseline to 6 months (SCS, 0.19 points; P2P, −0.12 points; DoD, −0.31 points; 95% CI, −0.52 to −0.11 points) and baseline to 12 months (SCS, 0.18 points; P2P, −0.53 points; DoD, −0.70; 95% CI, −0.92 to −0.49 points). Finally, at West Palm Beach, the P2P group had an increase in depressive symptoms, while the SCS group had a decrease in depressive symptoms from baseline to 6 months (SCS, −1.02 points; P2P, 0.04 points; DoD, 1.07 points; 95% CI, 0.48 to 1.65 points) and 12 months (SCS, −1.05 points; P2P, 0.09 points; DoD, 1.14; 95% CI, 0.51 to 1.77).

At 12 months, the P2P group at Rochester had a 2.8-point increase in the mental health component of health status and quality of life measure, and the SCS had a 2.3-point decrease, which was a significant DoD (5.1 points; 95% CI, 1.3-8.9 points) (eTable 4 in the Supplement). There was a significant difference in resilience at 6 months with the SCS group reporting a 0.45-point decrease, and the P2P group reporting a 0.10-point increase for a DoD of 0.56 points (95% CI, 0.32 to 0.79 points). At 12 months, the SCS group in Rochester reported a 0.02-point decrease in resilience, and the P2P group reported a 0.13-point increase, for a DoD of 0.15 points (95% CI, −0.09 to 0.38 points), which was not statistically significant. The P2P group at Rochester had a 0.86-point reduction in depressive symptoms compared with a 0.12-point increase in the SCS group at 12 months (DoD, −0.98 points; 95% CI, −1.58 to −0.39). Additionally, both SCS and P2P groups reported a decrease in self-efficacy from baseline to 12 months; however, the difference was larger in the SCS group than the P2P group (SCS, 0.31 points; P2P, 0.06 points; DoD: 0.24 points; 95% CI, 0.10 to 0.39 points). There were no differences in measures of emotional health at 6 months or 12 months at Los Angeles.

## Discussion

Our results were mixed and did not show a clear pattern of how receiving P2P support may improve or slow the decline of health and well-being in older adults compared with their peers who were receiving SCS. Our hypothesis that older adults in the P2P group would maintain a comparatively higher health status and quality of life and would have comparatively fewer depressive and anxiety symptoms than the SCS group was not supported by the data.

We were surprised that we did not find a pattern of association between participants who received P2P support and health and well-being. In a qualitative study conducted with a subset of participants in the P2P group, participants perceived P2P support services as highly valuable in helping them overcome the challenges of aging in place, particularly by improving mobility throughout the community and decreasing social isolation.^53^ Participants spoke of the value of their peer and how their peer “saved my life”^53^ by providing friendship and a means to get out of the house and interact with others. Additionally, previous studies suggest that P2P programs improve health and well-being in older adults^16,17,18,19,20^ and could even help older adults remain in their homes.^20^ Our findings are not consistent with the findings from our qualitative study or previous literature.

### Limitations

This study had several limitations. First, we wonder whether the results would have been different in a randomized trial of P2P support. Our community partners felt strongly that it was not appropriate to withhold services from older adults who sought out such services, so we did not conduct a randomized clinical trial. Because of this, we attempted to frequency-match participants by 3 demographic characteristics during recruitment to decrease the chances that there would be meaningful differences between the 2 groups at baseline.

Despite this matching, at baseline, participants who were in the SCS group had better physical and emotional health than those in the P2P group. Specifically, we think it is possible that significantly higher levels of social support in the SCS group at West Palm Beach and Los Angeles may have contributed to a null result. Rochester was the site with the fewest baseline differences and the only site with no significant difference in social support between the 2 groups at baseline. At Rochester, there were improvements in mental health, depressive symptoms, and self-efficacy within the P2P group compared with the SCS group. These patterns were more similar to previous literature that has highlighted how older adults in P2P support programs show decreases in anxiety and depressive symptoms.^17,18,19,20,21^ Social service agencies traditionally offer P2P programming to individuals with reduced access to transportation outside the house, higher levels of depression and anxiety, and overall worse health because they believe those individuals would be most likely to benefit from the P2P services. During recruitment, we attempted to enroll participants into the SCS who were similar to the P2P group. However, when we realized we had not succeeded and there were significant differences between the groups at baseline, we tried to account for these differences in the propensity score model. In future studies, it may be important to consider additional factors, such as social isolation, driving status, and social support,^54,55,56^ when enrolling older adults in a control group so that groups are more comparable at baseline.

A third limitation was that all of the participants in our study were not newly enrolled in the P2P program; some had been receiving P2P support for many years. It is possible that the effects of the P2P program are most noticeable during the initial months after enrollment. A fourth limitation is the observer effect. Simply being enrolled in the research study and the frequent contact that study team members had with participants in both the P2P and SCS groups may have negated the feelings of loneliness that some participants may have experienced before enrolling in the research study. Previous research has found that P2P programs specifically and befriending interventions more broadly improve social integration and personal relationships.^16,57^ Given anecdotes from the research coordinators who routinely heard from participants how much they enjoyed talking to them, the findings from the qualitative study,^53^ and the fact that loneliness in both SCS and P2P groups remained relatively stable over the 12-month research study, we think it is likely that many at-risk older adults experienced benefits from periodic social check-ins.

## Conclusions

The results of this study did not show associations between P2P services and improvements in health and well-being over time. It may be that P2P programs do not help maintain or slow the decline of health and well-being in older adults. It is also possible that our failure to account for important differences between the groups at baseline is why we were not able to show clear patterns of association. Well-conducted randomized trials with extended follow-up periods are needed to determine the association between P2P services and measures of health and well-being. This study highlights additional factors, such as social support, social isolation, and transportation, that would be important to account for at baseline when conducting future studies.

## Notes

Supplement. **eTable 1.** Outcome Measure, Tool Name, Time Point Measured, and Score Range and Meaning **eTable 2.** Measures of Health and Well-being Over Time and Difference of Differences at 6 and 12 Months Between P2P and SCS at Los Angeles, Inverse Probability Weighted Model (n = 75) **eTable 3.** Measures of Health and Well-being Over Time and Difference of Differences at 6 and 12 Months Between P2P and SCS at West Palm Beach, Inverse Probability Weighted Model (n = 177) **eTable 4.** Measures of Health and Well-being Over Time and Difference of Differences at 6 and 12 Months Between P2P and SCS at Rochester, Inverse Probability Weighted Model (n = 196) **eAppendix 1.** Protocol **eAppendix 2. **Analytical and Statistical Approaches
