# Supplementary material for: Peer-to-Peer Support and Changes in Health and Well-being in Older Adults Over Time
Source: JAMA Netw Open. 2021 Jun 15;4(6):e2112441. doi: 10.1001/jamanetworkopen.2021.12441 (PMC8207241; doi:10.1001/jamanetworkopen.2021.12441)
Supplement: Supplement. — eTable 1. Outcome Measure, Tool Name, Time Point Measured, and Score Range and Meaning eTable 2. Measures of Health and Well-being Over Time and Difference of Differences at 6 and 12 Months Between P2P and SCS at Los Angeles, Inverse Probability Weighted Model (n = 75) eTable 3. Measures of Health and Well-being Over Time and Difference of Differences at 6 and 12 Months Between P2P and SCS at West Palm Beach, Inverse Probability Weighted Model (n = 177) eTable 4. Measures of Health and Well-being Over Time and Difference of Differences at 6 and 12 Months Between P2P and SCS at Rochester, Inverse Probability Weighted Model (n = 196) eAppendix 1. Protocol eAppendix 2. Analytical and Statistical Approaches [file jamanetwopen-e2112441-s001.pdf]

Supplemental Online Content

Schwei RJ, Hetzel S, Kim KM, et al. Peer-to-peer support and changes in health and well-being in older adults over time. *JAMA Netw Open.* 2021;4(6):e2112441. doi:10.1001/jamanetworkopen.2021.12441

eTable 1. Outcome Measure, Tool Name, Time Point Measured, and Score Range and Meaning

eTable 2. Measures of Health and Well-being Over Time and Difference of Differences at 6 and 12 Months Between P2P and SCS at Los Angeles, Inverse Probability Weighted Model (n = 75)

eTable 3. Measures of Health and Well-being Over Time and Difference of Differences at 6 and 12 Months Between P2P and SCS at West Palm Beach, Inverse Probability Weighted Model (n = 177)

eTable 4. Measures of Health and Well-being Over Time and Difference of Differences at 6 and 12 Months Between P2P and SCS at Rochester, Inverse Probability Weighted Model (n = 196)

eAppendix 1. Protocol

eAppendix 2. Analytical and Statistical Approaches

This supplemental material has been provided by the authors to give readers additional information about their work.

© 2021 Schwei RJ et al. *JAMA Network Open.*

eTable 1. Outcome Measure, Tool Name, Time Point Measured, and Score Range and Meaning

**Outcome measure**	**Tool name**	**Time point measured**	**Score range and meaning**
Health status and quality of life	SF-12 Health Survey[37]	Baseline, 6 months, 12 months	• Ranged from 0 to 100<br>• 100 is perfect mental or physical health
Depressive Symptoms	Center for Epidemiological Studies Depress (CES-D)[38]	Baseline, 6 months, 12 months	• Ranged from 0 to 10<br>• 0 was no depressive symptoms<br>• 10 was 10 depressive symptoms
Anxiety Symptoms	Geriatric Anxiety Inventory (GAI)[39]	Baseline, 6 months, 12 months	• Ranged from 0 to 5<br>• 0 was no anxiety symptoms<br>• 5 was 5 anxiety symptoms
Loneliness	Short Scale for Measuring Loneliness in Large Surveys[40]	Baseline, 6 months, 12 months	• Ranged from 1 to 3<br>• 1 was less loneliness<br>• 3 was more loneliness
Self-Efficacy	General Self-Efficacy Scale(GSE)[41]	Baseline, 6 months, 12 months	• Ranged 1 to 4<br>• 1 was low self-efficacy<br>• 4 was high self-efficacy
Resilience	Brief Resilience Scale[42]	Baseline, 6 months, 12 months	• Ranged 1 to 5<br>• 1 was low resilience<br>• 5 was high resilience
Social Support	8 Item Medical Outcomes Social Support Survey (MOSS)[43]	Baseline, 6 months, 12 months	• Ranged 1 to 5<br>• 1 was high social support<br>• 5 was low social support
Self-Reported Disability-Activities of DailyLiving	Activities of Daily Living (ADLs)[7]	Baseline, 6 months, 12 months	• Ranged 0 to 6<br>• 0 could all activities independently<br>• 6 could complete no activitiesindependently
Self-Reported Disability-Instrumental Activities of Daily Living	Instrumental Activities of Daily Living (IADLs)[46]	Baseline, 6 months, 12 months	• Ranged 0 to 8<br>• 0 could complete all activitiesindependently<br>• 8 could complete no activitiesindependently
Self-Reported Disability-Basic Mobility and Strength	Rosow-Bresleau[47,48]	Baseline, 6 months, 12 months	• Ranged 0 to 3<br>• 0 could do all tasks<br>• 3 could do none of the tasks
Self-Reported Disability-Physical Function	Nagi[48,50]	Baseline, 6 months, 12 months	• Ranged 1 to 5<br>• 1 no difficulty with tasks<br>• 5 not able to do the tasks

eTable 2. Measures of Health and Well-being Over Time and Difference of Differences at 6 and 12 Months Between P2P and SCS at LosAngeles, Inverse Probability Weighted Model (n = 75)

	Group	Base	6 mo.	12 mo.	6 mo. Change from Baseline (95% CI)	6 mo. Difference of Differences (95% CI)	12 mo. Change from Baseline (95% CI)	12 mo. Difference of Differences (95% CI)
Health Status & Quality of Life								
Mental Health component	SCS†	44.6	46.2	48.1	1.6 (-1.8, 4.9)	-0.9	3.5 (0.3, 6.8)	-4.4
	P2P	50.7	51.4	49.8	0.7 (-3.7, 5.1)	(-6.4, 4.7)	-0.9 (-5.3, 3.6)	(-9.9, 1.1)
Physical Health Component	SCS†	40.7	42.3	35.3	1.5 (-1.6, 4.7)	-2.2	-5.4 (-8.5, -2.3)	5.9
	P2P	31.8	31.2	32.3	-0.6 (-4.8, 3.6)	(-7.4, 3.1)	0.5 (-3.7, 4.7)	(0.7, 11.1)[‖]
Depressive Symptoms[§]	SCS†	3.90	4.22	3.41	0.32 (-0.19, 0.83)	-0.33	-0.49 (-0.99, 0.02)	0.38
	P2P	3.77	3.76	3.66	-0.01 (-0.68, 0.67)	(-1.17, 0.52)	-0.11 (-0.79, 0.57)	(-0.47, 1.23)
Anxiety Symptoms[§]	SCS†	1.74	1.95	2.10	0.21 (-0.16, 0.59)	-0.23	0.36 (-0.01, 0.74)	-0.16
	P2P	1.73	1.72	1.93	-0.02 (-0.51, 0.48)	(-0.85, 0.40)	0.20 (-0.31, 0.71)	(-0.79, 0.46)
Loneliness[§]	SCS†	1.89	1.73	1.70	-0.16 (-0.32, 0.00)	0.05	-0.19 (-0.34, -0.03)	0.04
	P2P	1.79	1.68	1.65	-0.11 (-0.32, 0.10)	(-0.22, 0.32)	-0.14 (-0.36, 0.07)	(-0.22, 0.31)
Self-efficacy	SCS†	3.35	3.34	3.24	-0.01 (-0.14, 0.12)	0.07	-0.09 (-0.22, 0.04)	0.06
	P2P	3.27	3.33	3.26	0.06 (-0.12, 0.24)	(-0.15, 0.29)	-0.03 (-0.21, 0.15)	(-0.16, 0.28)
Resilience	SCS†	3.22	3.33	3.21	0.12 (-0.10, 0.33)	-0.14	0.00 (-0.21, 0.20)	-0.07
	P2P	3.49	3.47	3.41	-0.02 (-0.30, 0.26)	(-0.49, 0.22)	-0.08 (-0.36, 0.21)	(-0.42, 0.28)
Social Support[§]	SCS†	2.84	2.82	3.29	-0.02 (-0.26, 0.22)	-0.06	0.45 (0.21, 0.69)	-0.36
	P2P	3.47	3.39	3.56	-0.08 (-0.40, 0.24)	(-0.46, 0.34)	0.09 (-0.23, 0.42)	(-0.76, 0.05)
Self-reported Disability								
Activities of Daily Living[§]	SCS†	0.11	0.11	0.33	0.00 (-0.18, 0.18)	0.10	0.22 (0.04, 0.39)	-0.06
	P2P	0.44	0.54	0.59	0.10 (-0.14, 0.33)	(-0.20, 0.40)	0.16 (-0.08, 0.40)	(-0.36, 0.23)
Instrumental Activities of Daily Living	SCS†	0.97	1.17	1.31	0.19 (-0.33, 0.71)	0.21	0.34 (-0.18, 0.86)	0.11
	P2P	2.49	2.47	2.71	-0.02 (-0.71, 0.67)	(-0.20, 0.40)	0.23 (-0.47, 0.93)	(-0.76, 0.98)
Basic Mobility and Strength[§]	SCS†	1.32	1.21	1.59	-0.11 (-0.34, 0.11)	0.03	0.26 (0.04, 0.48)	-0.43
	P2P	2.16	2.08	2.00	-0.09 (-0.38, 0.21)	(-0.34, 0.40)	-0.17 (-0.47, 0.13)	(-0.80, -0.06)[‖]
Physical Function[§]	SCS†	2.39	2.34	2.28	-0.05 (-0.42, 0.33)	0.05	-0.11 (-0.48, 0.26)	0.00
	P2P	3.07	3.07	2.96	0.00 (-0.50, 0.50)	(-0.58, 0.67)	-0.12 (-0.62, 0.39)	(-0.63, 0.62)

[†] SCS: Standard community services group; [‡] P2P: Peer-to-peer group; [§] Variables where smaller numbers indicates better health; [‖] $p < 0.05$

© 2021 Schwei RJ et al. *JAMA Network Open.*

eTable 3. Measures of Health and Well-being Over Time and Difference of Differences at 6 and 12 Months Between P2P and SCS at West PalmBeach, Inverse Probability Weighted Model (n = 177)

	Group	Base	6 mo.	12 mo.	6 mo. Change from Baseline (95% CI)	6 mo. Difference of Differences (95% CI)	12 mo. Change from Baseline (95% CI)	12 mo. Difference of Differences (95% CI))
Health Status & Quality of Life								
Mental Health Component	SCS[†]	50.3	51.7	54.0	1.5 (-1.2, 4.1)	0.5	3.7 (0.9, 6.6)	-3.5
	P2P[‡]	46.2	48.2	46.5	1.9 (-1.0, 4.9)	(-3.5, 4.4)	0.2 (-2.8, 3.2)	(-7.6, 0.6)
Physical Health Component	SCS	43.4	43.4	43.3	0.0 (-2.0, 1.9)	0.2	-0.1 (-2.2, 2.0)	-0.7
	P2P	35.8	36.0	35.0	0.2 (-2.0, 2.3)	(-2.7, 3.1)	-0.8 (-3.0, 1.4)	(-3.7, 2.4)
Depressive Symptoms[§]	SCS	3.86	2.83	2.80	-1.02 (-1.41, -0.63)	1.07	-1.05 (-1.48, -0.62)	1.14
	P2P	3.66	3.71	3.75	0.04 (-0.40, 0.48)	(0.48, 1.65)[¶]	0.09 (-0.36, 0.54)	(0.51, 1.77)[¶]
Anxiety Symptoms[§]	SCS	1.46	1.05	0.99	-0.42 (-0.66, -0.17)	0.23	-0.47 (-0.75, -0.20)	0.75
	P2P	1.96	1.78	2.24	-0.19 (-0.46, 0.09)	(-0.14, 0.60)	0.28 (-0.01, 0.57)	(0.36, 1.15)[¶]
Loneliness[§]	SCS	1.34	1.33	1.40	0.00 (-0.09, 0.09)	0.06	0.06 (-0.04, 0.16)	0.10
	P2P	1.76	1.82	1.93	0.05 (-0.05, 0.15)	(-0.08, 0.19)	0.16 (0.06, 0.27)	(-0.04, 0.25)
Self-efficacy	SCS	3.45	3.53	3.51	0.08 (-0.04, 0.20)	-0.22	0.06 (-0.06, 0.19)	-0.16
	P2P	3.24	3.11	3.15	-0.14 (-0.26, -0.01)	(-0.39, -0.04)[‖]	-0.09 (-0.22, 0.04)	(-0.34, 0.02)
Resilience	SCS	3.64	3.83	3.82	0.19 (0.05, 0.33)	-0.31	0.18 (0.03, 0.32)	-0.70
	P2P	3.54	3.42	3.02	-0.12 (-0.27, 0.03)	(-0.52, -0.11)[‖]	-0.53 (-0.68, -0.37)	(-0.92, -0.49)[¶]
Social Support[§]	SCS	3.96	4.02	4.06	0.06 (-0.10, 0.22)	-0.04	0.10 (-0.07, 0.27)	0.07
	P2P	3.56	3.58	3.73	0.02 (-0.16, 0.19)	(-0.28, 0.20)	0.17 (-0.01, 0.35)	(-0.18, 0.31)
Self-reported Disability								
Activities of Daily Living[§]	SCS	0.23	0.41	0.35	0.19 (0.07, 0.31)	-0.01	0.13 (0.00, 0.26)	0.05
	P2P	0.45	0.62	0.63	0.18 (0.04, 0.31)	(-0.19, 0.17)	0.18 (0.05, 0.32)	(-0.14, 0.24)
Instrumental Activities of Daily Living[§]	SCS	0.35	1.17	0.92	0.82 (0.42, 1.22)	0.35	0.58 (0.13, 1.03)	-0.03
	P2P	1.31	1.78	1.92	0.47 (0.01, 0.92)	(-0.26, 0.96)	0.61 (0.14, 1.08)	(-0.68, 0.62)
Basic Mobility and Strength[§]	SCS	1.35	1.25	1.36	-0.10 (-0.27, 0.07)	-0.01	0.01 (-0.18, 0.20)	0.11
	P2P	1.97	1.86	2.09	-0.11 (-0.31, 0.08)	(-0.27, 0.25)	0.12 (-0.08, 0.32)	(0.16, 0.39)
Physical Function[§]	SCS	2.13	1.79	1.88	-0.33 (-0.59, -0.07)	-0.16	-0.25 (-0.53, 0.04)	0.28
	P2P	3.15	2.65	3.18	-0.49 (-0.79, -0.20)	(-0.55, 0.23)	0.04 (-0.26, 0.34)	(-0.13, 0.70)

[†]SCS: Standard community services group; [‡] P2P: Peer-to-peer group; [§]Variables where smaller numbers indicates better health; [‖] $p < 0.05$; [¶] $p < 0.001$

© 2021 Schwei RJ et al. *JAMA Network Open.*

eTable 4. Measures of Health and Well-being Over Time and Difference of Differences at 6 and 12 Months Between P2P and SCS at Rochester, Inverse Probability Weighted Model (n = 196)

Outcome	Group	Base	6 mo	12 mo	Change from Baseline (95% CI)	Difference of Differences (95% CI)	Change from Baseline (95% CI)	Difference of Differences (95% CI)
Health Status and quality of life								
Mental Health Component	SCS	53.7	52.7	51.5	-1.1 (.3.4, 1.3)	4.0 (0.1,	-2.3 (-4.7, .01)	5.1 (1.3, 8.9)[‖]
	P2P	51.8	54.7	54.5	2.9 (-0.1, 5.9)	7.8)[‖]	2.8 (-0.2, 5.8)	
Physical Health Component	SCS	40.8	38.3	42.0	-2.5 (-4.8, -0.1)	3.0	1.2 (-1.2, 3.5)	2.0
	P2P	37.7	38.2	40.8	0.5 (-2.5, 3.6)	(-0.8, 6.8)	3.2 (0.2, 6.1)	(-1.8, 5.8)
Depressive Symptoms[§]	SCS	3.58	3.01	3.70	-0.57 (-0.92, -0.22)	-0.25	0.12 (-0.25, 0.49)	-0.98
	P2P	3.94	3.12	3.07	-0.82 (-1.28, -0.36)	(-0.83, 0.33)	-0.86 (-1.33, -0.40)	(-1.58, -0.39)[‖]
Anxiety Symptoms[§]	SCS	1.53	1.13	1.07	-0.40 (-0.69, -0.12)	-0.08	-0.46 (-0.76, -0.16)	0.14
	P2P	1.53	1.05	1.22	-0.48 (-0.86, -0.11)	(-0.56, 0.39)	-0.32 (-0.70, 0.06)	(-0.34, 0.63)
Loneliness[§]	SCS	1.57	1.55	1.59	-0.02 (-0.13, 0.09)	-0.11	0.02 (-0.09, 0.13)	-0.03
	P2P	1.59	1.46	1.58	-0.13 (-0.27, 0.01)	(-0.29, 0.07)	-0.01 (-0.15, 0.13)	(-0.20, 0.15)
Self-efficacy	SCS	3.43	3.19	3.12	-0.23 (-0.32, -0.15)	0.29	-0.31 (-0.40, -0.22)	0.24
	P2P	3.28	3.33	3.21	0.05 (-0.06, 0.17)	(0.14, 0.43)[¶]	-0.06 (-0.18, 0.05)	(0.10, 0.39)[‖]
Resilience	SCS	3.71	3.26	3.70	-0.45 (-0.60, -0.31)	0.56	-0.02 (-0.16, 0.13)	0.15
	P2P	3.44	3.54	3.57	0.10 (-0.08, 0.29)	(0.32, 0.79)[¶]	0.13 (-0.05, 0.31)	(-0.09, 0.38)
Social Support[§]	SCS	3.79	3.61	3.74	-0.18 (-0.35, -0.01)	0.23	-0.05 (-0.22, 0.12)	0.00
	P2P	3.58	3.63	3.53	0.05 (-0.16, 0.27)	(-0.04, 0.50)	-0.05 (-0.26, 0.16)	(-0.26, 0.27)
Self-reported Disability								
Activities of Daily Living[§]	SCS	0.41	0.27	0.38	-0.14 (-0.31, 0.02)	0.03	-0.03 (-0.20, 0.13)	-0.16
	P2P	0.52	0.41	0.33	-0.11 (-0.32, 0.10)	(-0.24, 0.30)	-0.19 (-0.40, 0.02)	(-0.43, 0.11)
Instrumental Activities of daily living	SCS	1.19	1.28	1.00	0.09 (-0.24, 0.41)	-0.23	-0.20 (-0.54,0.15)	0.10
	P2P	1.56	1.88	1.26	0.32 (-0.11, 0.75)	(-0.77, 0.31)	-0.30 (-0.73, 0.13)	(-0.45, 0.66)
Basic Mobility and strength	SCS	1.60	1.21	1.48	-0.39 (-0.57, -0.21)	0.13	-0.12 (-0.31, 0.07)	0.17
	P2P	1.62	1.35	1.66	-0.27 (-0.50, -0.03)	(-0.17, 0.43)	0.05 (-0.20, 0.29)	(-0.14, 0.47)
Physical Function[§]	SCS	2.67	2.22	2.58	-0.45 (-0.74, -0.17)	0.36	-0.10 (-0.39, 0.20)	-0.03
	P2P	2.87	2.77	2.74	-0.09 (-0.47, 0.28)	(-0.11, 0.83)	-0.12 (-0.50, 0.25)	(-0.51, 0.45)

[†] SCS: Standard community services group; [‡] P2P: Peer-to-peer group; [§] Variables where smaller numbers indicates better health; [‖] $p < 0.05$; [¶] $p < 0.001$

© 2021 Schwei RJ et al. *JAMA Network Open.*

eAppendix 1. Protocol

The Effectiveness of Peer-to-Peer Community Support to Promote Aging in Place

Principal Investigator: Elizabeth Jacobs

Protocol Version Date: 10/30/2017

Coordinating Center: University of Wisconsin

Co-Investigators
Jane Mahoney- University of Wisconsin
KyungMann Kim-University of Wisconsin
Laura Pinsoneault- Alliance for Children and Families
Jennifer Frumer- Alpert Jewish Family and Children's Service
Katy Allen- Community Place
Paul Castro- Jewish Family Service of Los Angeles

Study Coordinator: Amy Amessoudji

Funding Sponsor: Patient Centered Outcomes Research Network (PCORI)

© 2021 Schwei RJ et al. *JAMA Network Open.*

Project Summary

Background: As highlighted on the American Association of Retired Persons website, the vast majority of older adults desire to live and age in their communities, yet they find it increasingly difficult because of "…how communities are constructed and [the lack of]…services offered." In response, many community-based organizations have initiated peer-to-peer support services to promote aging in place; they train and employ community older adults to provide support to other older adults through social visits and opportunities to access health and non-health related activities in the community. It is not yet clear how effective these services are.
Objectives: Our overall objective is to investigate the effectiveness of three community-designed and implemented peer-to-peer support programs to promote health and wellness in vulnerable older adult populations. Our Specific Aims are: (1) To compare the effectiveness of peer-to-peer community support in preventing hospitalization, emergency department (ED) use, and nursing home placement in an at-risk older adult population relative to receipt of standard community services and (2) To compare the effect of peer-to-peer community support on intermediary measures of health and overall wellness such as self-rated health, depression and anxiety relative to standard community services.
Methods: Building on an established relationship between the University of Wisconsin School of Medicine and Public Health and stakeholders at the Alliance for Children and Families and three community-based organizations, we will conduct a longitudinal comparative-effectiveness study of peer-to-peer community support in three diverse US communities. We will follow participants over 12 months and compare outcomes in 360 at-risk older adult community residents enrolled in a peer-to-peer support program to 360 at-risk older adult community residents receiving standard community services controlling for relevant socio-demographic and baseline factors that make them at-risk. Specifically, we will (1) compare annual rates of acute and long-term hospitalization, emergency department use, and institutionalization and (2) examine differences in self-reported health, depression, anxiety, activities of daily living in the group receiving peer support relative to the group receiving standard community services
Patient Outcomes: We have chosen our outcomes because they matter to older adults, the families, community organizations, health care providers and state insurers. None of these stakeholders want to see vulnerable older adults be hospitalized, place in a nursing home, or experience preventable intermediary outcomes such as depression that likely influence whether they can age in place. Given that 13% of the total US population was > 65 years of age in 2010 and more than 90% of them desire to age in place, the question of how to promote aging in place among older adults is highly relevant and important.

© 2021 Schwei RJ et al. *JAMA Network Open.*

Background and Significance

A. Background

In 2010, there were 40 million people age 65 and over in the United States, accounting for 13 percent of the total population. The older population in 2020 is projected to be twice as large as in 2000, growing from 35 million to 72 million and representing 20 percent of the total US population. Americans are living longer than ever and while most people age 65 and over report their health as good, very good or excellent, with age comes increased risk of certain diseases and disorders, such as heart disease, cancer, chronic lower respiratory diseases, stroke, diabetes, and infections such as influenza and pneumonia and their complications. These health conditions and the process of aging itself frequently lead to functional decline in older Americans. In 2009, about 41 percent of people age 65 and over enrolled in Medicare reported a functional limitation, with approximately 25 percent having difficulty with at least one activity of daily living such as eating, bathing, dressing, using the restroom independently and walking.

While this functional decline places them at risk for entering into nursing homes, most older adults live in the community and the vast majority want to continue to age in place, defined as "… being able to remain in one's current residence even when faced with increasing need for support." Among individuals age 65-74, about 97 percent reside in the community and nearly 90% of them desire to age in place. The question of how to effectively help older adults age in place is of great national import given the "greying" of the US population, the high prevalence of functional limitations among older adults, and this at-risk population's overwhelming desire to age in their own homes.

B. Significance

B.1 Addressing a Critical Gap in the Literature Despite the import of this question, most of the research to date has focused on what factors place older adults at risk of moving from community living to a nursing home rather than what can be done to prevent this transition. Known risk factors for long-term care placement in nursing homes include older age, type and number of chronic physical or mental health conditions, recent hospitalizations and/or ED visits, need for assistance with instrumental activities of daily living, social isolation, minority race/ethnicity, and economic disadvantage While this research helps us understand what factors should be targeted by interventions to promote aging in place, it does not provide evidence for what we can do to prevent nursing home placement.

© 2021 Schwei RJ et al. *JAMA Network Open.*

B.2 The Proposed Work is Novel and Promising Through our unique partnership we have the opportunity to do some of the first rigorous research on how community-initiated, based, and delivered interventions to provide support to older adults at risk for hospitalization and/or long-term institutionalization can help them continue to age in place*.* To date, the research on interventions to promote aging in place has mainly focused on how changes in the physical environment, such as the home environment, assistive technology, and home-base health services enhance the likelihood that older adults can age in place. Research focusing on the role that communities play in promoting aging in place is relatively new and important. As Greenfield and colleagues posit, "Beyond physical well-being, aging in place in community is also concerned with relationships, positive growth, life purpose and communal well-being. This idea is captured in Figure 1, which is a conceptual model of how community support service would promote aging in place; We use it to illustrate **our hypotheses** about how peer-to-peer support will promote aging in place. Specifically, we hypothesize that peer-to-peer support will promote social relationships and engagement with services through interaction with the peer and the peer's help with accessing social opportunities, health care, and other services within the community; this improved social milieu and access to opportunities and services will result in individuals having greater self-efficacy, social support, and access to services; this, in turn, will promote better physical and mental health and psychosocial well-being in individuals receiving peer support; all with the long-term outcome of promoting aging in place among older adults.

Figure 1: Community Supportive Service Programs Conceptual Framework

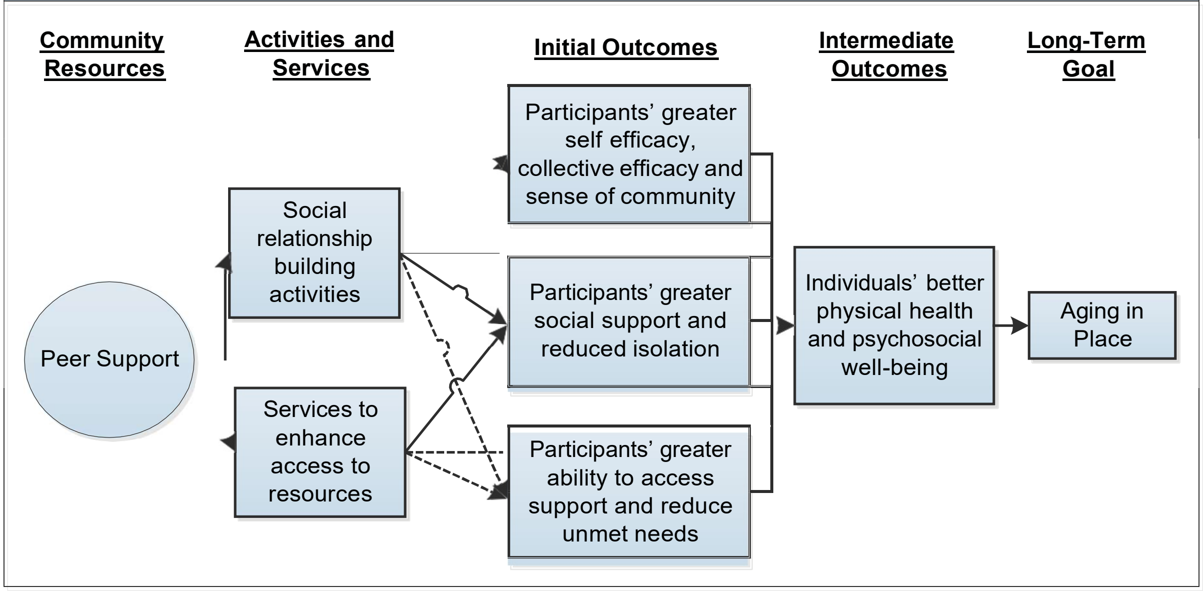

© 2021 Schwei RJ et al. *JAMA Network Open.*

Peer support, as conceptualized by Cindy-Lee Dennis (see Figure 2) is distinct from natural lay helpers, who have no or minimal training, or paraprofessionals, who have extensive training. Peer support is provided by trained lay individuals selected to give support within their peer group. In this study the peers are older adult volunteers who receive a small stipend and are given training to provide support to other older adults in their community through emotional support, social opportunities, encouragement and engendering of persistence and optimism, and facilitation of use of other community resources such as group activities or health care. Previous research supports the positive impact of this type of peer-support on health and health outcomes in a number of populations and conditions, including older adults. However, there are very few studies of general peer-support and the role it may play in the health and well-being of older adults.

Figure 2: Dennis Conceptual Distinctions of Peer Support

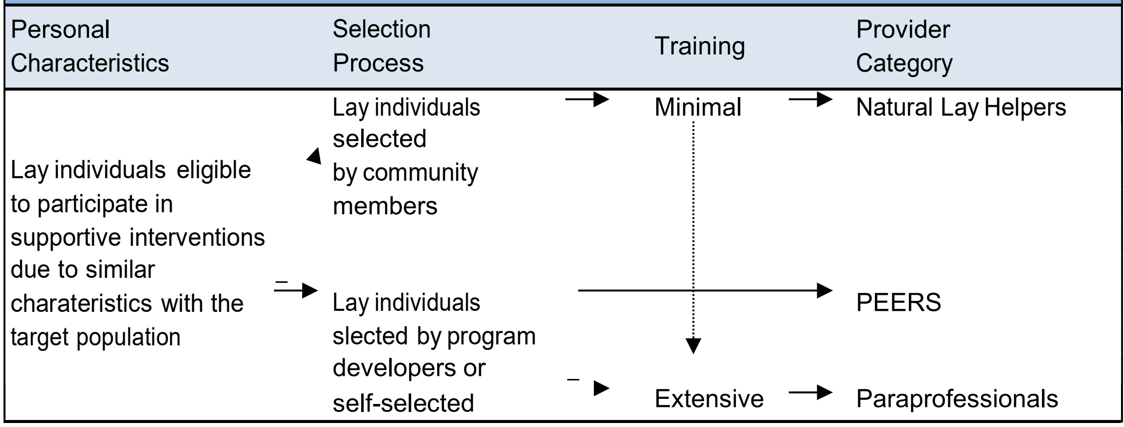

© 2021 Schwei RJ et al. *JAMA Network Open.*

Specific Aims

The vast majority of US adults older than 65 desire to age in their communities, yet they find it increasingly difficult because of "…how communities are constructed and [the lack of]…services offered." In response, many community-based organizations have initiated peer-to-peer support services to promote aging in place; they train older adults to provide support to their peers through social visits and opportunities to access health and non-health related activities in their shared community. It is not yet clear how effective these services are. **Our overall objective** is to compare the effectiveness of peer-to-peer support programs in preventing utilization of acute health care and nursing home services in older adult populations at risk for needing these services and promoting health and wellness in this population relative to older adults receiving standard community services. **Our Specific Aims are**:

(1) To compare the effectiveness of peer-to-peer community support in preventing hospitalization, emergency department (ED) use, and nursing home placement in an at-risk older adult population relative to standard community services. *H1:* Older adults in the peer-to-peer support group will have lower rates of hospitalization, ED visits, and nursing home placement compared to the standard service group.

(2) To compare the effect of peer-to-peer community support on intermediary measures of health and wellness such as self-rated health, depression, and anxiety relative to standard community services. *H1:* Older adults in the peer-to-peer support group will have higher self-rated health and overall wellness and less depression and anxiety compared to the standard service group.

Building on an established relationship between the University of Wisconsin School of Medicine and Public Health and stakeholders at The Alliance for Children and Families and three community-based organizations, we will conduct a longitudinal comparative-effectiveness study of peer-to-peer community support in three diverse US communities. We will follow participants over 12 months and compare outcomes in 360 at-risk older adult community residents enrolled in a peer-to-peer support program to 360 at-risk older adult community residents receiving standard community services, controlling for relevant socio-demographic and baseline factors.

We have chosen our outcomes because they matter to older adults, their families, communities, health care providers, and insurers. None of these stakeholders want to see vulnerable older adults be hospitalized, enter a nursing home, or experience preventable outcomes like depression that may impact successful aging in place. Given that 13% of the US population was $\geq$ 65 years of age in 2010 and more than 90% of them desire to age in place, the question of how to promote aging in place among older adults through "real world", easily disseminated and implemented interventions is highly relevant and important.

© 2021 Schwei RJ et al. *JAMA Network Open.*

Research Design and Methods

C.1 **Choice of Study Design** We will accomplish our aims by conducting a longitudinal comparative-effectiveness study in which at-risk older adult study participants in three communities across the US are followed for 12-months. Using a mixed experimental and quasi-experimental design, we will compare outcomes in those receiving peer-to-peer community support to those who are receiving standard community services. We have chosen a 12-month period to follow older adults so as to maximize the chance we will be able to see a change in our outcomes over time within the constraint of the maximum 3-year project period.

The strengths of our design include a multiple community study, strengthening generalizability; a study of an established intervention that has already shown feasibility and reach in many communities; well-defined comparator groups; real-world inclusion criteria, exemplary of a pragmatic trial; and methods to account for potential confounders in our analysis. An additional strength is that we can randomize at risk older adults meeting the criteria for receiving peer-support to participate in the peer-to-peer support program (Intervention) or to receive standard community services (Control) at Alpert Jewish Family & Children's Services of West Palm Beach (AJFCS of Palm Beach). We are able to randomize at this site because the stakeholders and community are willing to have their participants randomized, and they recently received a grant to expand their program, allowing randomization to be integrated into how they implement their soon to be expanding peer-to-peer support program. We cannot randomize at our other two sites, Jewish Family Services of Los Angeles (JFS of LA) and Community Place of Greater Rochester because their stakeholders and communities do not find randomization acceptable; the reality is that they have long waiting lists for their programs and do not want to move frail, older adults from their position on these lists. While it might be simpler and "cleaner" to conduct only an observational study across all three sites, we have chosen to use a mix of a randomized trial and quasi-experimental (group comparison) trial rather than a quasi-experimental trial alone because (1) randomization at one site is stronger than randomization at none and (2) we can use data at the randomized site to compare to the other two sites to check the validity of our findings in the observational sites (see C.3.f Analytic Plan). We are also able to account for this design difference across the sites in our analysis (see C.3.f Analytic Plan).

C.2 **Setting, Standard Community Services, & Peer-to-Peer Community Support**

C.2.a. Setting The peer-to-peer community support efforts we will study are taking place in three diverse communities in the US: Los Angeles, CA, West Palm Beach, FL, and Rochester, NY. The community-based organizations that provide these efforts are Jewish Family Services of Los Angeles (JFS of LA), Alpert Jewish Family & Children's Services of West Palm Beach (AJFCS of Palm Beach), and Community Place of Greater Rochester. The UW and Alliance investigators chose to collaborate with these three organizations for several reasons: (1) They are very motivated to study the value of peer-to-peer support in helping their older adult community members to successfully age in place, (2) Their peer-to-peer programs are established, large, and similar in design and training, (3) The communities and members they serve are racially and ethnically diverse (see C.3.c.iii. Sample Diversity).

C.2.b Standard Community Services Each organization serves more than ten thousand older adults a year through a variety of services, including their peer-to-peer support services. All three are well-established in their communities, providing aging services since at least the early 1980s and have established peer-support programs that have been in place for 10 or more years. They predominantly serve low-income older adults who are struggling to live independently in their communities due to economic strain or declining health. Many of their clients are socially isolated and live alone and have a continuum of functional impairments that impact their daily living and self-care. The standard aging services offered at each site include

health, wellness, socialization and enrichment activities, case management and counseling, resource referrals, food pantry and meal delivery. Older adults in the peer-support program (described in detail below) will also have access to these same standard services but have the added support of a peer who helps with social engagement, encouragement and, potentially, transportation to these resources.

C.2.c Peer-to-Peer Community Support

C.2.c.i. Standard Elements All three study sites have large peer-to-peer support programs (serving between 200-400 frail older adults) with standardized core elements (See Table 3). Core program elements include the same program objective, standard definition of who qualifies for peer-to-peer support, the mechanism by which older adults are referred for consideration for peer-support, core elements of training programs for the older adults who volunteer to provide the peer support, and monthly in-service trainings for all volunteers once trained, weekly hours that volunteers spend providing support, and provision of small stipends for volunteers. The current number of peer volunteers at the sites ranges from 45-95; As they find their role very rewarding, there is very little peer turn-over; the vast majority of peers volunteer for years in this role, until they themselves start requiring services.

Sites train their volunteers in the same array of means and methods for supporting their elderly clients. Peer volunteers then use these methods while tailoring their support to the individual needs of the clients they serve. For example, some clients need more support than others. Further, as we describe in **E. Patient Population** we will have a diverse study population. While the standard elements of the intervention are the same across all sites, despite this diversity, sites add custom elements to meet their unique community needs. For example, training modules on how to address the unique needs of and barriers faced by limited English proficient older adults. This is in keeping with norms of implementation of evidence-based interventions in health care. Differences across organizations must be accommodated in order to maximize the effectiveness of interventions in the real world; as long as fidelity to key intervention elements is maintained.[28]

C.3.d Study Population

C.3.d.i Exclusion/Inclusion Criteria To be included in the study, older adults must be ≥65 years of age, live independently in their community year-round, and meet the community defined criterion for receiving peer-to-peer support: have one or more of the following risk factors low-income (at or below poverty level) or a fixed income that barely meets their living expenses, social and/or familial isolation, chronic illness, and/or in need of frequent community services or resources. We will exclude adults who are younger than 65, have cognitive impairment (TICS ≤ 30),[32] or who would be unlikely to receive peer-to-peer support services for at least a year. Individuals who are unlikely to receive at least a year of services include those who need short-term help after a surgery and are likely to return to full functioning and those planning to transition to nursing home care or move away. Potential participants who express an interest in participating will complete the 8-item, 10-minute TICS screening and will be asked about their intentions to move or enter long term care so as to only include eligible older adults. Those older adults who only need services for a short time will not be referred to the study.

To be included in the peer-support group older adults must also be enrolled in this program within their community and have an assigned peer volunteer. To be included in the standard community services group the older adult must meet the qualifications for receiving peer-to-peer support from the community organization, but not be enrolled because they have been randomized to the standard community group, in the case of AJFCS of Palm Beach, or they are on a waiting list for entrance into the peer-support group. Because of the need and limited

© 2021 Schwei RJ et al. *JAMA Network Open.*

Table 3: Core Elements of the Peer to Peer Community Support Intervention	
Goal	• To promote successful aging in place among frail elderly
Duration of Peer to Peer Relationship	• From enrollment until patient transitions to more advanced care or dies
Target Population	• Older adults at risk for a decline in health or placement in long-term care
Referral Proccess	• Self-referral or referal by case-managers from the community organization or local health care organizations
Volunteer Selection	• Adults older than 65 that are able to dedicate 20 hours a week to Peer Support<br>• Need to commit for a minimum of one year
Volunteer Training	Initial Training<br>• 10-20 Hours<br>Training Modules<br>• Developing a peer-to-peer support relationship<br>• Importance of compainionship<br>• Basic health and emotional health needs of at-risk older adults<br>• How to provide emotional support<br>• An overview of services provided by the organization and by the community and how to access them<br>• Trouble shooting particular issues that might arise in a relationship<br>Monthly In-Service Training<br>• 1-2 Hours<br>• Relevant Topic
Expectations of Volunteers	• Attend all trainings<br>• Attend at least 60% of monthly inservice trainings<br>• Provide a minimum of 20 hours of peer support<br>• Contact assigned peers on a regular basis
Peer Client Load per Volunteer	• Minimum of 2 to a maximum of 10
Shaped to Meet the Local Community Needs	• Each program adds training on particular issues and/or community resources unique to the community they serve

resources for providing peer-to-peer support at JFS of LA and Community Place of Greater Rochester and the fact that most older adults using peer-support services do not disenroll from it, the transition from waiting list to peer-to-peer support is an infrequent event; 1-2 older adults per month. If an older adult is admitted to peer-to-peer support from the control group, they will continue to be counted as being in the control group in an intention to treat analysis.

C.3.d.ii *Peer-to-peer Support or Standard Community Services Group Membership* As stated previously, how an individual gets assigned to one of these two study groups will vary because we will be able to randomize individuals at one of the study sites. We anticipate that at least 240 of the 400 older adults identified by AJFCS of Palm Beach as qualifying for the peer-to-peer support will be willing to participate and be randomized to the peer-to-peer support (n=120) and standard community service (n=120) groups. Older adults at this site will be randomized to entering immediately into the peer-to-peer support program or waiting for 6 months prior to

© 2021 Schwei RJ et al. *JAMA Network Open.*

entering into the program; 6 months was the longest period that the AJFCS felt comfortable making older adults wait for these services. This also confers some design advantage in that it will allow us to also test whether or not peer-support is "dose-related" = more effective when provided for 12 months compared to 6 months (see C.3.f Analytic Plan). At each of the other two sites we will include 120 older adults in the peer-to-peer support group and 120 in the standard community services group for a total intervention group size of 360 (120 from each site) and 360 in the control group (120 from each site; see Figure 3). At JFS of LA and Community Place of Greater Rochester, older adults already receiving the peer-to-peer support services will be included in the peer-to-peer support group as will any participants who begin receiving the services during the study enrolment period (month 6 to 18; see Appendix C: Detailed Project Plan and Timeline) for a total of 120 participants in the peer support group at each site. Participants who meet criteria to be enrolled in peer-to-peer support services but are on the waiting list will be matched to those receiving the services by age, gender, and race/ethnicity to make the groups as comparable as possible. Data from both JFS of LA and Community Place of Greater Rochester support the fact that 1,000 or more of their clients would meet criteria to receive peer-support services so there will be rich pool of eligible individuals who could volunteer to enroll in the standard community support to reach the target size of 120 at-risk older adults at each site.

Figure 3: Study Design at AJFCS of Palm Beach

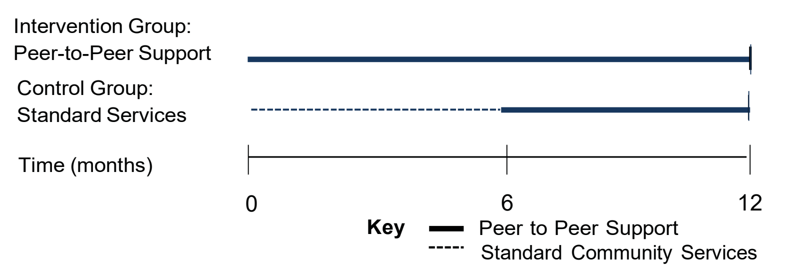

C.3.e Study Outcomes & Measures To meet our first two aims we will (1) compare annualized rates of hospitalization, ED use, and nursing home placement and (2) examine the changes in self-reported health, depression, anxiety, and other measures of well-being in the group receiving peer-to-peer support compared to the group receiving standard community services from baseline to the end of study enrolment. We describe each of our outcome and additional study measures in detail below. *Measures have been translated and used in Spanish and have been shown to be valid or have high reliability in Spanish.*[33-43]

C.3.e.i Main Outcome Measures--Health Care Utilization & Rates of Nursing Home Placement
We will collect data on number of hospitalizations and ED visits via survey at 3 different time points for each individual: baseline, 6 months, and 12 months (see Figure 4 under ***C.3.f. Data Collection Procedures***). We will start by asking four questions measuring health care utilization from the Stanford Disease Self-Management Study.[42] These questions ask about how many times respondents have visited an ED, went to a hospital emergency room, stayed in the hospital overnight, and how many nights total they spent in a hospital, all in the past 6 months. These questions have been shown to have high test-retest reliability in an older population of individuals with chronic disease[42] and are correlated with actual documented usage.[44] We will also ask them if they have benefited from visiting nurse or home health aid services and the duration and frequency of use of those services. To facilitate recall of this information, we will provide each participant with a calendar with color-coded stickers that they can place on the days each month they went to the emergency department and/or were hospitalized or stayed the hospital. We will ask them to refer to this calendar when being surveyed; if they have lost it will be noted in the data collection instrument and data will be based on their recall. Regarding nursing home placement we will ask them if they have moved to a nursing home or plan to in the next 4 weeks.

Validation of Health Care Utilization Data

© 2021 Schwei RJ et al. *JAMA Network Open.*

Because the validity of this data may be impacted by recall bias, we have planned to validate the data by randomly selecting 10% of participants in each group (peer-to-peer support and standard community services) at each site and ask for their permission to access their medical record information from their physicians and health care organizations. If they agree they will sign a release of medical information form to release data for the year they were enrolled in the study. Specifically, hospitals and physician offices will be contacted to verify data regarding dates of participant reported ED visits, and/or hospitalizations. This is technique used frequently when recall bias poses a data problem, such as in completing diet diaries, and can improve data validity.[45] In addition, Dr. Mahoney has successfully used this method of validating recall of almost identical health care utilization data in a past study of 500 older adults in 4 counties in the State of Wisconsin; more than 90% of providers supplied the heath care data requested.

C.2.e.ii. Intermediary Measures of Health, Well-being, and Social Support We will collect data on health status, quality of life, symptoms of depression and anxiety, loneliness, resiliency, general self-efficacy, and social support using validated, standardized instruments as outlined below. We have chosen these measures because they are of strong interest to our stakeholders and, as indicated in our conceptual model (see B.1 Addressing a Critical Gap in the Literature), they are known risk factors for increased health care risk factors and utilization,[13] and nearly all have been validated for use in diverse populations and in both English and Spanish languages. As with the health care and nursing home data, we will collect this data via survey at 3 different time points for each individual: baseline, 6 months, and 12 months (see Figure 4 under **C.3.f. Data Collection Procedures**).

Health Status & Quality of Life: The 12-item Short Form Health Survey, the SF-12, is commonly used to measure physical and mental health status and the impact of this status on an individual's quality of life. It has been specifically tested and shown to be reliable and valid for use with older adults with slightly modified scoring.[46-48] It includes a 1-item question on self-rated health and several items on mental and physical health and how much physical and mental health issues have impacted the respondent's life over the past 4 weeks.

Depressive Symptoms: The 10-item version of the Center of Epidemiologic Studies-Depression (CES-D) scale is used to assess depressive symptoms. The CES-D was developed for the assessment of depressive symptoms in the general population[49] and focuses on symptoms experienced in a recent time period, usually the last 1 or 2 weeks. The CES-D is a widely used measure of depressive symptoms in epidemiologic studies of older adults, and its psychometric properties are well-established.[50] The shorter 10-item form was developed specifically to reduce participant burden in older adults, and its correspondence to the original version has been established previously.[51]

Anxiety Symptoms: The 5-item version of the Geriatric Anxiety Inventory, the Geriatric Anxiety Inventory-Short Form (GAI-SF) was developed and validated for use to measure anxiety symptoms in older populations. It has been shown to be reliable (Cronbach's alpha = 0.81) and it is both sensitive (87%) and specific (86%) for detecting Generalized Anxiety Disorder in geriatric populations.[52]

Loneliness: will be measured using the Short Scale for Measuring Loneliness in Large Surveys. This is a 3-item measure that has been shown to be a valid and reliable measure of loneliness in surveys of older adults and to predict future mortality in this population.[53] It consists of 3 items in which individuals are asked how often (hardly ever, some of the time, or often) they feel a lack of companionship, feeling left out, and feeling isolated from others.

© 2021 Schwei RJ et al. *JAMA Network Open.*

Self-efficacy: The General Self-Efficacy Scale (GSE)[54] is a 10-item scale that has been validated to assess an individual's general sense of perceived self-efficacy including perceived ability to cope with daily hassles and stressful life events. In samples from 23 nations, Cronbach's alphas have averaged in the high 0.80s and validity has been documented in a number of correlations studies demonstrating that greater self-efficacy, as measured using this tool, is significantly related to positive emotions and optimism. [55]

Resilience: The Brief Resilience Scale (BRS) is a 6-item instrument designed to measure the ability of individuals to bounce back from stress.[56] It includes items such as "I tend to bounce back quickly after hard times." It has been documented to be reliable and valid in a variety of populations, including an older adult population with chronic illness

Social Support: will be measured with the 8-item Medical Outcomes Study Social Support Survey. This instrument covers 4 domains: emotional/informational support, tangible or instrumental support, positive social interactions, and affection.[57] It was carefully constructed based on sound theoretical theory, originally for use in chronically ill adults. It has been used in numerous studies to date and shown to be universally acceptable in multiple populations, including the elderly.[58-60]

C.2.e.iii Additional Measures to Account for Other Factors that Might Influence Our Outcomes
We will include several additional measures in our analysis so that we can account for other factors that might influence our measured outcomes. They include socio-demographic characteristics of the study participants, their current living situation and social support, behavioral risk factors for health care use (e.g. smoking), health and functional status, cognitive status, previous health care utilization, and how often and what kind of services are provided through peer-support and standard community services. We describe how we will measure these variables in a standardized way below. In addition, we will include an indicator for study site in our analyses to account for differences in study design across sites (a randomized, controlled trial for AJFCS and a matched, quasi-experimental study in JFS of LA and Community Place) in our analyses (see C.3.g.i. Analysis Plan below).

Socio-demographic Variables: Age is assessed via self-reported date of birth, which is used to compute age at time of interview. Race/ethnicity is classified according to the 1990 U.S. Census categories, including non-Hispanic White, Hispanic White, African-American, or other. Marital status is ascertained in 5 categories (never married, married, widowed, separated, divorced) and we will ask about current living arrangements (alone, with partner, with other family member, and/or other caregiver). Information on socioeconomic status will be derived from questions about current income and total household income. Educational attainment is recorded in terms of years of formal schooling completed.

Self-reported disability is based on the following four measures: Katz measure of limitations in Activities of Daily Living (ADL); the Duke Older Americans Resources and Services (OARS) measure of Instrumental Activities of Daily Living (IADL); Rosow-Breslau measure of tasks requiring basic mobility and strength; and the Nagi measure of basic physical functions. The 6-item measure of ADL disability assesses the ability to perform basic self-care functions (e.g., eating, bathing, dressing) without help.[61,62] The 10-item OARS measure of IADL disability focuses on more complex tasks typically required to live independently in the community (e.g., preparing meals, shopping for groceries).[63] Each task is rated on a 5-point degree of difficulty scale. The 3-item Rosow-Breslau measure assess the ability to perform 3 basic tasks (walking half a mile; climbing a flight of stairs; doing heavy household chores).[64] The Nagi measures assesses difficulties in performing basic physical activities (e.g., pulling or pushing large objects; crouching and kneeling) on a 5-point scale (range, no difficulty – unable to do).[65,66] Summary

© 2021 Schwei RJ et al. *JAMA Network Open.*

scores will be constructed for each measure by adding scores across individual items. These measures have shown good to excellent reliability and reproducibility in older populations[67,68] and are well-established and well-accepted in gerontologic research. Although the measures are inter-related, each measure characterizes a distinct aspect of the broader concept of disability.[69,70]

Health care utilization variables We will ask 4 questions from the Medical Expenditure Panel Survey (MEPS) from the Agency for Healthcare Research and Quality, which is a well-established set of large-scale surveys that collect data on the specific health services that Americans use and how frequently they use them, among other things.[71] The questions will ask about access to a usual source of care, the source of care (community clinic, hospital-based clinic, etc), whether or not they see one doctor on a regular basis at that source of care, and if so how many times they have seen that doctor in the past 6 months.[71] For the outpatient visits we will ask about total number of visits during this time period and the number of visits to a usual source of care. We will also ask if they have previously been in a long-term care facility as well as plans or intent in the next 6 months to enter an such a facility.

Behavioral and Physical Health Variables: Measures and indicators of health behaviors include cigarette smoking, alcohol consumption, physical activity, and weight. Structured questions on smoking and alcohol consumption were adopted from the Established Populations for Epidemiologic Studies of The Elderly (EPESE) studies.[72] Physical activity is assessed using a measure derived from the 1985 Health Interview Survey, with some adaptation to make the questions more relevant for older adults.[73,74] This measure assesses participation in 10 physical activities during the past 2 weeks (e.g., walking, gardening, dancing), and for each activity both the frequency of participation in the last 2 weeks and duration are assessed. Body mass index (BMI) is used as a measure of relative weight, and calculated as self-reported weight in kilograms divided by height in meters squared. We will measure cognitive status using the short form of the Telephone Interview for Cognitive Status (TICS-m). It is an 8-item measure that asks questions about date, address, counting backward, among other things that has been shown to be a reliable, valid measure of cognitive status in older adults.[32]

Chronic Health Conditions & Medication Use: Information on prevalent chronic conditions is obtained through self-report, using standardized questions administered at each in-person interview on physician-diagnosed diseases derived from the EPESE.[72] Relevant questions are related to myocardial infarction, stroke, diabetes, cancer, high blood pressure, hip fracture, and Parkinson's disease. In addition to self-report of physician-diagnosed conditions, information on the time since first diagnosis of the condition and current medication use for each condition is also obtained at each interview.

Peer-to-peer Support Variables: We will ask participants how often they have had contact with their peer in the last 4 weeks and to choose the types of services they have provided them from a list (e.g. ride to appointment, social support visit, help with cooking, etc).

<u>*C.2.e.iv. Concern for Respondent Burden;*</u> As in any survey study, we have been conscious of keeping respondent burden at a minimum by choosing the shortest, validated measures that capture our measures of interest. We believe the number of measures (N=23) we have chosen are not overly burdensome and feasible for completion by older adult study participants based on Dr. Jacobs current National Institute on Aging funded study in partnership with the successful longitudinal Chicago Health and Aging Project Study; in this study more than 95% of older adults (average age = 74 years) are successfully followed every 3 years, complete a survey that is three times as long and includes the same or similar items.[55,75,76] In addition, we pilot-tested

© 2021 Schwei RJ et al. *JAMA Network Open.*

our measurement battery (see the survey in Appendix A: Measures) by interviewing older adult stakeholders and found it took 30-40 minutes to complete.

C.3.f. Data Collection Procedures At risk older adults will be followed for 12 months with structured study contact (see Figure 4) and survey data collected in-person. They will be contacted by the study coordinator from their community site and, if they are interested in participating after they hear a description of the study, a visit for the initial consent and baseline survey will be scheduled. We anticipate the initial visit will take 45-60 minutes and will consist of: review of the study and what it entails, informed consent process, completion of the baseline interview, and review of the calendar data collection procedure for them to note their health care utilization data. They will receive a gift card for $50 at the end of this interview. They will then be visited at 5.5-6 months to collect healthcare utilization and intermediary outcome data, address, and contact information updates. At 12 months, they will participate in another study visit in which data from all the same variables (except baseline demographic data) will be collected. They will receive a $50 for completing final data collection. To mitigate potential reporting bias by the older adults in the peer-support group that might occur because their peer supporter might remind them to mark down the dates of health care utilization, we will call all participants at 3 and 9 months just to remind them to continue to mark down this data on their calendars (Figure 4)

Figure 4: Timeline of Study Participation, Contact, and Data Collected for Individual Study Participants

Recruited into Study — (12 Months) — End of Study Participation

Time	0 Months	3 Months	6 Months	9 Months	12 Months
Type of Contact	In-person Interview	Reminder Phone Call	Phone Interview	Reminder Phone Call	In-person Interview
Data Collected	All Study Variables	None	Healthcare Utilization and Intermediary Variables	None	All Study Variables Except Demographics

Research Team and Environment

As we describe below, project team members bring expertise regarding multisite trials, aging, geriatrics, biostatistics, community-engaged research, delivery and evaluation of community based interventions, provision of client-centered aging services, including peer-support to older adults, and the needs of older adults wanting to age in place and the family members who support them. A major strength of this team is that it includes both experienced researchers and stakeholders and that it will be led by both a researcher and a stakeholder.

The lead researcher, **Elizabeth A Jacobs, MD MPP**, is Associate Professor in the Departments of Medicine and Population Health Sciences, Associate Vice Chair for Health Services Research in the Department of Medicine, and an integral member of the Health Innovation Program (HIP), all within the UWSMPH. She is a practicing general internist at a Federally Qualified Health Center and a recognized expert on the conduct of research investigating racial and ethnic disparities in health and receipt of health care and the impact of interventions to reduce them on the amount and quality of healthcare received.[82-95] She has conducted several large-scale investigations on this topic and has received research funding from numerous Federal and foundation sources. She is currently the Principal Investigator on an R01 from the National Institute on Aging entitled “Trust in Healthcare and Racial Disparities in an Aging Population. In addition, her potential for contributing to the development of patient-centered care has been recognized by PCORI and she has served as chair of one of PCORI’s pilot research prioritization committees and is a member of the Addressing Disparities advisory panel. Her record of successful, innovative, patient-centered, community-based research and experience in multi-site, intervention, and aging research make her well suited to lead the team of investigators in partnership with stakeholders to successfully complete this work. The lead stakeholder and co-investigator, **Laura Pinsoneault, MS** is Director of Evaluation and Research for The Alliance. She conducts research on national initiatives and programs that seek to

© 2021 Schwei RJ et al. *JAMA Network Open.*

strengthen the leadership capacity of high performing community-based organizations and what helps them reach their impact goals in the areas of poverty, education, health and economic security. She has been conducting research in the human services sector for 18 years and has extensive experience in working with stakeholder groups and evaluating and disseminating community-based interventions. She represents the stakeholder perspective of community service organizations; her expertise in studying and performing evaluations of human services interventions and efforts of community-based organizations make her an excellent co-lead with Dr. Mahoney.

Additional aging research and biostatistical expertise is provided by two UWSMPH faculty members: named PI **Jane Mahoney, MD,** board-certified Internist and Geriatrician and Professor of Geriatrics and **KyungMann Kim, PhD**, Professor of Biostatistics and Statistics. Dr. Mahoney is Executive Director of the Wisconsin Institute for Healthy Aging (WIHA, www.wihealthyaging.org), a non-profit organization that works with the Aging Network and health care organizations to disseminate evidence-based prevention programs to older adults in Wisconsin and across the US. She is also Principal Investigator of the Community-Academic Aging Research Network, an NIH-funded network for community-based participatory research with Wisconsin's aging network. She has been funded extensively to conduct aging research and conducted clinical trials of community-based multifactorial interventions to decrease falls, and conducted dissemination research on evidence-based falls prevention programs.[96-105] As Executive Director of WIHA, she has overseen the dissemination and high-fidelity implementation of 4 evidence-based programs in Wisconsin (Chronic Disease Self-Management Program, Diabetes Self-Management Program, Stepping On falls Prevention Program, Powerful Tools for Caregivers to over 3000 older adults in Wisconsin each year), and one evidence-based program nationally (Stepping On to 16 other states). Through this work, she has also developed expertise in evaluating intervention implementation and fidelity. Dr. Kim has almost 30 years of experience in the design, conduct, monitoring, analysis and reporting of clinical trials in cancer, cardiovascular disease, AIDS/HIV, autoimmune disease, neurology and bone disease.[106-112] He is currently serving as the principal statistician for a community-intervention trial known as "Healthy Children Strong Families: American Indian Communities Preventing Obesity (HCSF-2)" funded by R01 HL114912-01 from the National Institutes of Health.

The team also includes 13 additional stakeholders: **Linda Nguyen, MAPP** Director of Civic Engagement for The Alliance; **Katy Allen, BA**, Director of the Aging Services Department at the Community Place of Greater Rochester (NY); **Paul Castro JD**, chief executive officer of Jewish Family Service of Los Angeles (JFS of LA); **Jenni Frumer, MSEd, MSW**, Associate Executive Director at the Alpert Jewish Family & Children's Service and The Levine Jewish Residential and Family Services in West Palm Beach, Florida (AJFCS of Palm Beach); **Vivian Saur, MSW,** Chief Program Officer of JFS of LA; **Eli Veitzer, BA,** Director of Strategic Initiatives and Business Development at JFS of LA.

© 2021 Schwei RJ et al. *JAMA Network Open.*

Data and Safety Monitoring Plan:

Data Collection: Data will be recorded directly into a structured electronic database developed on Datstat. The information collected will be self-coding whenever possible. Brief instructions for completing measures will be available to the data collectors on Datstat. Additional instructions, including those for standard data formats (e.g., dates, justification, and zero-filling), will be included in the Protocol Instruction Manual Data will be collected and recorded at scheduled protocol-defined data-collection points. Protocol-specified important, such as death or transition to a nursing home will be reported as they occur.

Training: Dr. Jacobs and the UW Team, with input from Ms. Pinsoneault, will develop a program for training data collection at each study site prior to study initiation. Topics to be covered include the following: Study design, procedures and data collection requirements; electronic data collection form completion, including data correction; adverse event reporting; source documentation requirements; human subjects regulatory requirements; and communications. The importance of minimizing the loss of the major outcome data will be emphasized during the training sessions.

Site Monitoring: We are planning site initiation visits prior to subject enrollment at each site. In addition we are planning to audit 10% of cases, and will conduct site audits for key fidelity elements. Findings from site monitoring visits will be reviewed to discuss possible mechanisms for improvement and the need for additional or targeted site monitoring at specific sites.

Data Management: Datstat, will be used for electronic data collection and entry at the sites. The UW team will define a study-specific data dictionary using an interactive development and testing process. Datstat provides a secure, web-based, flexible system with an intuitive interface for sites to enter data and have real time validation rules with automated data type and range checks at the time of data entry. Datstat offers easy data manipulation with audit trails and reporting for reporting, monitoring, and querying records, and an automated export mechanism to common statistical packages, including SAS. Study participants will be assigned a Study ID at the time of consent to the study. It will be a unique 8 digit number that includes a check digit.

Reporting Adverse Events: In the unlikely case of a breach in confidentiality study staff will notify their site PI and the site PI will notify Dr. Mahoney and Ms. Pinsoneault. Dr. Mahoney will then notify the UW IRB and will follow standard reporting procedures. During training study staff will be provided with an information sheet with referral information that can be provided to patients in the case of emotional upset. If a patient becomes upset, the study staff will encourage the participant to contact their primary care physician, a social worker or Dr. Mahoney. Study staff will inform their site PI of any emotional upset that occurs and site PIs will use best judgement in determining if it is necessary to notify Ms. Pinsoneault and Dr. Mahoney.

© 2021 Schwei RJ et al. *JAMA Network Open.*

Data and Record Keeping

The researchers at the University of Wisconsin will be responsible for managing all of the study data. All data will be collected via in person or telephone interview.

Face-to-face verbal surveys will be conducted in a private room. All data collected will be directly entered onto a laptop computer and then when the survey is complete before the research staff leaves the participant's home, all data will be uploaded directly to Datstat server.

The Datstat server is housed in a state-of-the-art data center managed by the UW SMPH

Patient names will not be associated with the survey data but all participants will be given a study ID number. Study ID number will be associated with patient name on a cross-walk file stored in the Datstat tracking system.

Paper medical records will be stored in a locked cabinet in a locked room and only study staff will be able to access these documents.

Identifiers will be removed from the data used for analysis but we'll retain the written consent forms with signatures for 7 years after the end of the research (to protect against possible charges of research fraud)

© 2021 Schwei RJ et al. *JAMA Network Open.*

References

1. Farber N, Shinkle D. Aging in Place: A State Sruvey of Livability Policies and Practicies. Available at: http://www.aarp.org/home-garden/livable-communities/info-11-2011/Aging-In-Place.html. Accessed April 9, 2013.
2. Gerace A. AARP: Seniors' Desire to Age in Place Faces Serious Challenges. Available at: http://seniorhousingnews.com/2011/12/20/aarp-seniors-desire-to-age-in-place-faces-serious-challenges/. Accessed April 12, 2013.
3. Selby J. Inroducing a New PCORI Research Funding Initiative-Large Pragmatic Clincal Trials. Available at: http://www.pcori.org/blog/introducing-a-new-pcori-research-funding-initiative-large-pragmatic-trials/. Accessed January 17, 2014.
4. Patient-Centered Outcomes Research Institute. Pre-Announcement: Large Pragmatic Trials to Evaluate Comparative Clinical Effectiveness. Available at: http://www.pcori.org/funding-opportunities/funding-announcements/pre-announcement-pragmatic-studies-to-evaluate-comparative-clinical-effectiveness/. Accessed January 17, 2014.
5. Markides KS, Ray LA, Branch A, Espino DV. Hispanic Established Populations for the Epidemiologic Study of the Elderly (HEPESE) Wave 5, 2004-2005. Available at: http://www.icpsr.umich.edu/icpsrweb/ICPSR/studies/25041. Accessed January 17, 2014.
6. Federal Interagency Forum on Aging Related Statistics. *Older Americans 2012 Key Indicators of Well-Being*; 2012.
7. Greenfield EA. Using ecological frameworks to advance a field of research, practice, and policy on aging-in-place initiatives. *Gerontologist.* 2012;52(1):1-12.
8. Depp CA, Jeste DV. Definitions and predictors of successful aging: a comprehensive review of larger quantitative studies. *Am J Geriatr Psychiatry.* 2006;14(1):6-20.
9. Morley JE. Aging in place. *J Am Med Dir Assoc.* 2012;13(6):489-492.
10. Robison J, Shugrue N, Porter M, Fortinsky RH, Curry LA. Transition from home care to nursing home: unmet needs in a home- and community-based program for older adults. *J Aging Soc Policy.* 2012;24(3):251-270.
11. Patient-Centered Outcomes Research Institute. Funding Announcements: Improving Heatlh Care Systems. Available at: http://www.pcori.org/assets/PFA-Improving-Healthcare-Systems.pdf. Accessed April 9, 2013.
12. Vasunilashorn S, Steinman BA, Liebig PS, Pynoos J. Aging in place: evolution of a research topic whose time has come. *J Aging Res.* 2012;2012:120952. PMCID: 3227373
13. Greenfield EA, Scharlach A, Lehning AJ, Davitt JK. A conceptual framework for examining the promise of the NORC program and Village models to promote aging in place. *J Aging Stud.* 2012;26(3):273-284.
14. Dennis CL. Peer support within a health care context: a concept analysis. *Int J Nurs Stud.* 2003;40(3):321-332.
15. Buman MP, Giacobbi PR, Jr., Dzierzewski JM, Aiken Morgan A, McCrae CS, Roberts BL, Marsiske M. Peer volunteers improve long-term maintenance of physical activity with older adults: a randomized controlled trial. *J Phys Act Health.* 2011;8 Suppl 2:S257-266. PMCID: 3181088
16. Carroll DL, Rankin SH, Cooper BA. The effects of a collaborative peer advisor/advanced practice nurse intervention: cardiac rehabilitation participation and rehospitalization in older adults after a cardiac event. *J Cardiovasc Nurs.* 2007;22(4):313-319.
17. Coull AJ, Taylor VH, Elton R, Murdoch PS, Hargreaves AD. A randomised controlled trial of senior Lay Health Mentoring in older people with ischaemic heart disease: The Braveheart Project. *Age Ageing.* 2004;33(4):348-354.

© 2021 Schwei RJ et al. *JAMA Network Open.*

18. Hildingh C, Fridlund B. Participation in peer support groups after a cardiac event: a 12-month follow-up. *Rehabil Nurs.* 2003;28(4):123-128.
19. Chapin RK, Sergeant JF, Landry S, Leedahl SN, Rachlin R, Koenig T, Graham A. Reclaiming joy: pilot evaluation of a mental health peer support program for older adults who receive medicaid. *Gerontologist.* 2013;53(2):345-352.
20. Goldman LM. The Friendly Companion Program. *Journal of Gerontological Social Work.* 2003;40(1-2):123-133.
21. Kim SH. [Effects of a volunteer-run peer support program on health and satisfaction with social support of older adults living alone]. *J Korean Acad Nurs.* 2012;42(4):525-536.
22. MacIntyre I, Corradetti P, Roberts J, Browne G, Watt S, Lane A. Pilot study of a visitor volunteer programme for community elderly people receiving home health care. *Health Soc Care Community.* 1999;7(3):225-232.
23. Wilson A. Improving life satisfaction for the elderly living independently in the community: care recipients' perspective of volunteers. *Soc Work Health Care.* 2012;51(2):125-139.
24. Wishart L, Macerollo J, Loney P, King A, Beaumont L, Browne G, Roberts J. "Special steps": an effective visiting/walking program for persons with cognitive impairment. *Can J Nurs Res.* 2000;31(4):57-71.
25. Adamy J, McGinty T. The Crushing Cost of Care. Available at: http://online.wsj.com/article/SB10001424052702304441404577483050976766184.html. Accessed April 8, 2013.
26. Activities of Daily Living. Available at: http://www.investopedia.com/terms/a/adl.asp. Accessed April 12, 2013.
27. Houser A. Nursing Homes Fact Sheet. Available at: http://assets.aarp.org/rgcenter/il/fs10r_homes.pdf. Accessed April 12, 2013.
28. Kilbourne AM, Neumann MS, Pincus HA, Bauer MS, Stall R. Implementing evidence-based interventions in health care: application of the replicating effective programs framework. *Implement Sci.* 2007;2:42. PMCID: 2248206
29. Bellg AJ, Borrelli B, Resnick B, Hecht J, Minicucci DS, Ory M, Ogedegbe G, Orwig D, Ernst D, Czajkowski S. Enhancing treatment fidelity in health behavior change studies: best practices and recommendations from the NIH Behavior Change Consortium. *Health Psychol.* 2004;23(5):443-451.
30. Breitenstein SM, Gross D, Garvey CA, Hill C, Fogg L, Resnick B. Implementation fidelity in community-based interventions. *Res Nurs Health.* 2010;33(2):164-173. PMCID: 3409469
31. Lee CY, August GJ, Realmuto GM, Horowitz JL, Bloomquist ML, Klimes-Dougan B. Fidelity at a distance: assessing implementation fidelity of the Early Risers prevention program in a going-to-scale intervention trial. *Prev Sci.* 2008;9(3):215-229.
32. Fong TG, Fearing MA, Jones RN, Shi P, Marcantonio ER, Rudolph JL, Yang FM, Kiely DK, Inouye SK. Telephone interview for cognitive status: Creating a crosswalk with the Mini-Mental State Examination. *Alzheimers Dement.* 2009;5(6):492-497. PMCID: 2783323
33. Babler J, Schwarzer R. Evaluación de la autoeficacia: Adaptación española de la escala de autoeficacia general [Measuring generalized self-beliefs: A Spanish adaptation of the General Self-Efficacy scale]. . *Ansiedad y Estrés.* 1996;2(1):1-8.
34. Black SA, Ray LA, Angel RJ, Espino DV, Miranda M, Markides KS. Resource Book of the Established Populations for Epidemiologic studies of the Elderly. *National Archive for Computerized Data on Aging*. Ann Arbor, MI; 2003:1-5.
35. Costa Requena G, Salamero M, Gil F. [Validity of the questionnaire MOS-SSS of social support in neoplastic patients]. *Med Clin (Barc).* 2007;128(18):687-691.
36. Fortmann AL, Gallo LC, Roesch SC, Mills PJ, Barrett-Connor E, Talavera GA, Elder JP, Matthews KA. Socioeconomic status, nocturnal blood pressure dipping, and

psychosocial factors: a cross-sectional investigation in Mexican-American women. *Ann Behav Med.* 2012;44(3):389-398. PMCID: 3767761

37. Heilemann MV, Lee K, Kury FS. Psychometric properties of the Spanish version of the Resilience Scale. *J Nurs Meas.* 2003;11(1):61-72.
38. Hu J, Wallace DC, Tesh AS. Physical activity, obesity, nutritional health and quality of life in low-income Hispanic adults with diabetes. *J Community Health Nurs.* 2010;27(2):70-83. PMCID: 2864930
39. Markides KS, Stroup-Brenham CA, Black SA, Satish S, Perkowski LC, Ostir G. The Health of Mexican American Elderly: Selected Findings from the Hispanic EPESE. In: Wykly ML, Ford AB, eds. *Serving Minority Elders in the 21st Century*. New York, New York: Springer Publishing Company Inc.; 1999:72-90.
40. Marquez-Gonzalez M, Losada A, Fernandez-Fernandez V, Pachana NA. Psychometric properties of the Spanish version of the Geriatric Anxiety Inventory. *Int Psychogeriatr.* 2012;24(1):137-144.
41. Raji MA, Ostir GV, Markides KS, Goodwin JS. The interaction of cognitive and emotional status on subsequent physical functioning in older mexican americans: findings from the Hispanic established population for the epidemiologic study of the elderly. *J Gerontol A Biol Sci Med Sci.* 2002;57(10):M678-682.
42. Stanford Chronic Disease Self-Management Study. Psychometrics. In: Lorig K, Stewart A, Ritter P, Gonzalez V, Laurent D, Lynch J, eds. *Outcome Measures for Health Education and other Health Care Interventions*. Thousand Oaks, CA: Sage Publications; 1996:24-25.
43. Gude Ruiz R, Calvo Mauri JF, Carrasco Lopez FJ. [The Spanish version and pilot study of a telephone test of cognitive status for evaluation and screening in dementia assessment and follow-up]. *Aten Primaria.* 1994;13(2):61-66.
44. Ritter PL, Stewart AL, Kaymaz H, Sobel DS, Block DA, Lorig KR. Self-reports of health care utilization compared to provider records. *J Clin Epidemiol.* 2001;54(2):136-141.
45. Pepe MS. Inference using surrogate outcome data and a validation sample. *Biometrika.* 1992;79(2):355-365.
46. Cernin PA, Cresci K, Jankowski TB, Lichtenberg PA. Reliability and validity testing of the short-form health survey in a sample of community-dwelling African American older adults. *J Nurs Meas.* 2010;18(1):49-59.
47. Resnick B, Parker R. Simplified scoring and psychometrics of the revised 12-item Short-Form Health Survey. *Outcomes Manag Nurs Pract.* 2001;5(4):161-166.
48. Ware JE, Jr., Kosinski M, Gandek B, Aaronson NK, Apolone G, Bech P, Brazier J, Bullinger M, Kaasa S, Leplege A, Prieto L, Sullivan M. The factor structure of the SF-36 Health Survey in 10 countries: results from the IQOLA Project. International Quality of Life Assessment. *J Clin Epidemiol.* 1998;51(11):1159-1165.
49. Randloff LS. The CES-D Scale A Self-Report Depression scale for Research in the General Population. *Applied Psychological Measurement.* 1977;1(3):385-401.
50. Hertzog C, Van Alstine J, Usala PD, Hultsch DF, Dixon R. Measurement properties of the Center for Epidemiological Studies Depression Scale (CES-D) in older populations. *Psycholgical Assessment.* 1990;2(1):64-72
51. Kohout FJ, Berkman LF, Evans DA, Cornoni-Huntley J. Two shorter forms of the CES-D (Center for Epidemiological Studies Depression) depression symptoms index. *J Aging Health.* 1993;5(2):179-193.
52. Byrne GJ, Pachana NA. Development and validation of a short form of the Geriatric Anxiety Inventory--the GAI-SF. *Int Psychogeriatr.* 2011;23(1):125-131.
53. Hughes ME, Waite LJ, Hawkley LC, Cacioppo JT. A Short Scale for Measuring Loneliness in Large Surveys: Results From Two Population-Based Studies. *Res Aging.* 2004;26(6):655-672. PMCID: 2394670

54. Schwarzer R, Jerusalem M. The General Self-Efficacy Scale (GSE). Available at: http://userpage.fu-berlin.de/~health/engscal.htm. Accessed April 9, 2013.
55. Bienias J, Beckett L, Bennett D, Wilson R, Evans D. Design of the Chicago Healthy Aging Project (CHAP). *J Alzheimers Dis.* 2003;5(5):349-355.
56. Smith BW, Dalen J, Wiggins K, Tooley E, Christopher P, Bernard J. The brief resilience scale: assessing the ability to bounce back. *Int J Behav Med.* 2008;15(3):194-200.
57. Moser A, Stuck AE, Silliman RA, Ganz PA, Clough-Gorr KM. The eight-item modified Medical Outcomes Study Social Support Survey: psychometric evaluation showed excellent performance. *J Clin Epidemiol.* 2012;65(10):1107-1116.
58. Hays RD, Sherbourne CD, Mazel RM. *User's Manual for the Medical Outcomes Study (MOS) Core Measures of Health-Related Quality of Life*. Santa Monica. CA: RAND Corporation; 1995.
59. Sherbourne C. Social functioning: social activity limitations measure. In: Stewart A, Jr. JW, eds. *Measuring functioning and well-being: the Medical Outcomes Study approach*. Durham, NC: Duke University Press; 1992:172-181.
60. Sherbourne CD, Stewart AL. The MOS social support survey. *Soc Sci Med.* 1991;32(6):705-714.
61. Branch LG, Jette AM. A prospective study of long-term care institutionalization among the aged. *Am J Public Health.* 1982;72(12):1373-1379. PMCID: 1650559
62. Katz S, Down TD, Cash HR, Grotz RC. Progress in the development of the index of ADL. *The Gerontologist.* 1970;10(1):20-30.
63. Fillenbaum GG, Smyer MA. The development, validity, and reliability of the OARS multidimensional functional assessment questionnaire. *J Gerontol.* 1981;36(4):428-434.
64. Rosow I, Breslau N. A Guttman health scale for the aged. *J Gerontol.* 1966;21(4):556-559.
65. Nagi SZ. An epidemiology of disability among adults in the United States. *Milbank Mem Fund Q Health Soc.* 1976;54(4):439-467.
66. Smith LA, Branch LG, Scherr PA, Wetle T, Evans DA, Hebert L, Taylor JO. Short-term variability of measures of physical function in older people. *J Am Geriatr Soc.* 1990;38(9):993-998.
67. Hoeymans N, Wouters ER, Feskens EJ, van den Bos GA, Kromhout D. Reproducibility of performance-based and self-reported measures of functional status. *J Gerontol A Biol Sci Med Sci.* 1997;52(6):M363-368.
68. Tager IB, Swanson A, Satariano WA. Reliability of physical performance and self-reported functional measures in an older population. *J Gerontol A Biol Sci Med Sci.* 1998;53(4):M295-300.
69. Brock DB, Lemke JH, Branch LG, Evans DA, Berkman LF. Mortality and physical functioning in epidemiologic studies of three older populations. *J Aging Soc Policy.* 1994;6(3):21-37.
70. Wolinsky FD, Callahan CM, Fitzgerald JF, Johnson RJ. Changes in functional status and the risks of subsequent nursing home placement and death. *J Gerontol.* 1993;48(3):S94-101.
71. Agency for Healthcare Research and Quality. *MEPS HC-147 2011 Full Year Consolidated Data File*. Rockville, MD; 2013.
72. Cornoni-Huntley J, Ostfeld AM, Taylor JO, Wallace RB, Blazer D, Berkman LF, Evans DA, Kohout FJ, Lemke JH, Scherr PA, et al. Established populations for epidemiologic studies of the elderly: study design and methodology. *Aging (Milano).* 1993;5(1):27-37.
73. McPhillips JB, Pellettera KM, Barrett-Connor E, Wingard DL, Criqui MH. Exercise patterns in a population of older adults. *Am J Prev Med.* 1989;5(2):65-72.
74. Moss AJ, Parsons VL. *Current estimates from the National Health Interview Survey, United States, 1985*. Washington DC: National Center for Health Statistics; 1986.

© 2021 Schwei RJ et al. *JAMA Network Open.*

75. Delgado J, Jacobs EA, Lackland DT, Evans DA, de Leon CF. Differences in blood pressure control in a large population-based sample of older African Americans and non-Hispanic whites. *J Gerontol A Biol Sci Med Sci.* 2012;67(11):1253-1258. PMCID: 3474465
76. CHAP: Chicago Health and Aging Project. Collaboration. Available at: https://chapstudy.org:8443/CHPWebApps/collaboration.jsp. Accessed April 10, 2013.
77. National Research Council. *The prevention and treatment of missing data in clinical trials*. Washington DC: National Academies Press; 2010.
78. Ghosh D, Lin DY. Semiparametric analysis of recurrent events data in the presence of dependent censoring. *Biometrics.* 2003;59(4):877-885.
79. Gearing R, El-Bassel N, Ghesquiere A, Baldwin S, Gillies J, Ngeow E. Major ingredients of fidelity: a review and scientific guide to improving quality of intervetnion research implementation. *Clin Psychol Rev.* 2011;31(1):79-88.
80. Robb SL, Burns DS, Docherty SL, Haase JE. Ensuring treatment fidelity in a multi-site behavioral intervention study: implementing NIH Behavior Change Consortium recommendations in the SMART trial. *Psychooncology.* 2011;20(11):1193-1201. PMCID: 3198011
81. Oakley A, Strange V, Bonell C, Allen E, Stephenson J. Process evaluation in randomised controlled trials of complex interventions. *BMJ.* 2006;332(7538):413-416. PMCID: 1370978
82. Betancourt JR, Jacobs EA. Language barriers to informed consent and confidentiality: the impact on women's health. *J Am Med Womens Assoc.* 2000;55(5):294-295.
83. Diamond LC, Wilson-Stronks A, Jacobs EA. Do hospitals measure up to the national culturally and linguistically appropriate services standards? *Med Care.* 2010;48(12):1080-1087.
84. Gadon M, Balch GI, Jacobs EA. Caring for patients with limited English proficiency: the perspectives of small group practitioners. *J Gen Intern Med.* 2007;22 Suppl 2:341-346. PMCID: 2078540
85. Jacobs E, Chen AH, Karliner LS, Agger-Gupta N, Mutha S. The need for more research on language barriers in health care: a proposed research agenda. *Milbank Q.* 2006;84(1):111-133. PMCID: 2690153
86. Jacobs EA. Patient centeredness in medical encounters requiring an interpreter. *Am J Med.* 2000;109(6):515.
87. Jacobs EA, Diamond LC, Stevak L. The importance of teaching clinicians when and how to work with interpreters. *Patient Educ Couns.* 2010;78(2):149-153.
88. Jacobs EA, Karavolos K, Rathouz PJ, Ferris TG, Powell LH. Limited English proficiency and breast and cervical cancer screening in a multiethnic population. *Am J Public Health.* 2005;95(8):1410-1416. PMCID: 1449374
89. Jacobs EA, Lauderdale DS. Receipt of cancer screening procedures among Hispanic and non-Hispanic health maintenance organization members. *Cancer.* 2001;91(1 Suppl):257-261.
90. Jacobs EA, Lauderdale DS, Meltzer D, Shorey JM, Levinson W, Thisted RA. Impact of interpreter services on delivery of health care to limited-English-proficient patients. *J Gen Intern Med.* 2001;16(7):468-474. PMCID: 1495243
91. Jacobs EA, Sadowski LS, Rathouz PJ. The impact of an enhanced interpreter service intervention on hospital costs and patient satisfaction. *J Gen Intern Med.* 2007;22 Suppl 2:306-311. PMCID: 2078550
92. Jacobs EA, Shepard DS, Suaya JA, Stone EL. Overcoming language barriers in health care: costs and benefits of interpreter services. *Am J Public Health.* 2004;94(5):866-869. PMCID: 1448350

© 2021 Schwei RJ et al. *JAMA Network Open.*

93. Jacobs JM, Maaravi Y, Cohen A, Bursztyn M, Ein-Mor E, Stessman J. Changing profile of health and function from age 70 to 85 years. *Gerontology.* 2012;58(4):313-321.
94. Karliner LS, Jacobs EA, Chen AH, Mutha S. Do professional interpreters improve clinical care for patients with limited English proficiency? A systematic review of the literature. *Health Serv Res.* 2007;42(2):727-754. PMCID: 1955368
95. Jacobs E. *Assessing the Impact of Provider-Patient Language Barriers on Health Care Costs and Quality*. Chicago, IL: Office of Minority Health (OMH), Office of Public Health and Science (OPHS), U.S. Department of Health and Human Services, and Cosmos Corporation for the study; 2003.
96. Callen BL, Mahoney JE, Wells TJ, Enloe M, Hughes S. Admission and discharge mobility of frail hospitalized older adults. *Medsurg Nurs.* 2004;13(3):156-163; quiz 164.
97. Gleason CE, Gangnon RE, Fischer BL, Mahoney JE. Increased risk for falling associated with subtle cognitive impairment: secondary analysis of a randomized clinical trial. *Dement Geriatr Cogn Disord.* 2009;27(6):557-563. PMCID: 2742559
98. Gray SL, Mahoney JE, Blough DK. Medication adherence in elderly patients receiving home health services following hospital discharge. *Ann Pharmacother.* 2001;35(5):539-545.
99. Mahoney J. Improving recovery of function after hospitalization. *J Am Geriatr Soc.* 2000;48(12):1730-1731.
100. Mahoney JE, Glysch RL, Guilfoyle SM, Hale LJ, Katcher ML. Trends, risk factors, and prevention of falls in older adults in Wisconsin. *WMJ.* 2005;104(1):22-28.
101. Mahoney JE, Palta M, Johnson J, Jalaluddin M, Gray S, Park S, Sager M. Temporal association between hospitalization and rate of falls after discharge. *Arch Intern Med.* 2000;160(18):2788-2795.
102. Mahoney JE, Sager MA, Jalaluddin M. New walking dependence associated with hospitalization for acute medical illness: incidence and significance. *J Gerontol A Biol Sci Med Sci.* 1998;53(4):M307-312.
103. Mahoney JE, Sager MA, Jalaluddin M. Use of an ambulation assistive device predicts functional decline associated with hospitalization. *J Gerontol A Biol Sci Med Sci.* 1999;54(2):M83-88.
104. Mahoney JE, Shea TA, Przybelski R, Jaros L, Gangnon R, Cech S, Schwalbe A. Kenosha County falls prevention study: a randomized, controlled trial of an intermediate-intensity, community-based multifactorial falls intervention. *J Am Geriatr Soc.* 2007;55(4):489-498.
105. Przybelski R, mahoney J. Best clinical practice models to reduce falls. In: Alexander N, Hausdorf J, eds. *Evaluation and Management of Gait Disorders*. New York: Marcel Dekker; 2005.
106. Frank D, Jumonville A, Loconte NK, Schelman WR, Mulkerin D, Lubner S, Richter K, Winterle N, Wims MB, Dietrich L, Winkler JM, Volk M, Kim K, Holen KD. A phase II study of capecitabine and lapatinib in advanced refractory colorectal adenocarcinoma: A Wisconsin Oncology Network study. *J Gastrointest Oncol.* 2012;3(2):90-96. PMCID: 3397648
107. Gee J, Bailey H, Kim K, Kolesar J, Havighurst T, Tutsch KD, See W, Cohen MB, Street N, Levan L, Jarrard D, Wilding G. Phase II open label, multi-center clinical trial of modulation of intermediate endpoint biomarkers by 1alpha-hydroxyvitamin D2 in patients with clinically localized prostate cancer and high grade pin. *Prostate.* 2013.
108. Li N, Park DH, Sun J, Kim K. Semiparametric transformation models for multivariate panel count data with dependent observation process. *Can J Stat.* 2011;39(3):458-474. PMCID: 3368240
109. Rocque G, Onitilo A, Engel J, Pettke E, Boshoven A, Kim K, Rishi S, Waack B, Wisinski KB, Tevaarwerk A, Burkard ME. Adjuvant therapy for HER2+ breast cancer: practice,

© 2021 Schwei RJ et al. *JAMA Network Open.*

perception, and toxicity. *Breast Cancer Res Treat.* 2012;131(2):713-721. PMCID: 3329126
110. Todem D, Hsu WW, Kim K. On the efficiency of score tests for homogeneity in two-component parametric models for discrete data. *Biometrics.* 2012;68(3):975-982.
111. Yang RK, Kalogriopoulos NA, Rakhmilevich AL, Ranheim EA, Seo S, Kim K, Alderson KL, Gan J, Reisfeld RA, Gillies SD, Hank JA, Sondel PM. Intratumoral hu14.18-IL-2 (IC) induces local and systemic antitumor effects that involve both activated T and NK cells as well as enhanced IC retention. *J Immunol.* 2012;189(5):2656-2664. PMCID: 3424361
112. Wisinkski K, Faeber A, Wagner S, Havighurst T, McElroy, Kim K, Bailey H. Predictors of Willingness to Participate in Window-of-Opportunity Breast Trials. *Clinc Med Res.* in press.

1. National Research Council. *The prevention and treatment of missing data in clinical trials.* Washington DC: National Academies Press; 2010.
2. Ghosh D, Lin DY. Semiparametric analysis of recurrent events data in the presence of dependent censoring. *Biometrics.* 2003;59(4):877-885.

eAppendix 2. Analytical and Statistical Approaches

Overall Approach

Our primary comparative effectiveness analyses were based on 2-sample tests. These included the *t* test, Wilcoxon rank sum test, Fisher exact test, and chi-square test. Our choice of analytic method depended on whether the outcome measures were continuous or categorical. We compared the aim 1 end points-namely, of UC, ED, and hospital use individually and as a composite-between the 2 groups using 2-sample tests at a 2-tailed significance level of a = .05. Similarly, we compared the aim 2 end points-health status and QOL, depressive symptoms, anxiety symptoms, loneliness, self-efficacy, resilience, social support, ADL, IADL, basic mobility and strength, and physical function-between the 2 groups using the same 2-sample tests and significance level.

We used a 2-tailed significance level of a = .05 without adjustment for multiplicity of outcomes and testing. *P* values in an observational study should be carefully interpreted because of the many strong assumptions that are required but are not verifiable, such as the assumption of there being no unmeasured confounders.

After comparison, the baseline characteristics of the 2 groups {P2P and SCS} were not similar despite our efforts to frequency-base match them according to age, sex, and race/ethnicity. Therefore, we used a propensity score analytic method to account for these differences at baseline.

© 2021 Schwei RJ et al. *JAMA Network Open.*

Building a Propensity Score Model

To build a propensity score model, we first conducted a logistic regression using group status {P2P vs SCS} as the outcome and baseline characteristics as covariates to understand the relationship between group status and these characteristics. Baseline characteristics included measures of previous health care use, baseline health status, baseline well-being, and demographic information. We selected baseline patient characteristics that were associated with being in the P2P group individually at the $P < .15$ level in this logistic regression to include in the final propensity score model. We then used the final propensity score model to estimate a propensity score for each participant. We weighted our final regression models using the inverse propensity score as a means for adjusting for factors associated with group status.[52]

To assess the adequacy of our propensity score approach, we examined a histogram plot of the inverse probability weights for each group as shown in Figure 3. The 2 study groups {orange is P2P and turquoise is SCS}, when looked at side by side, appear to be very similar. The weights distribution does not appear to differ meaningfully between the 2 groups, demonstrating that the propensity score model used in the analyses "balanced" differences between the P2P and SCS groups. Essentially, this figure gave us confidence that any differences we observed in the main outcomes between the 2 study groups were due to study group assignment and not due to underlying differences in measured baseline characteristics between the 2 groups.

© 2021 Schwei RJ et al. *JAMA Network Open.*

Figure 3. Propensity Model Weights by Study Group

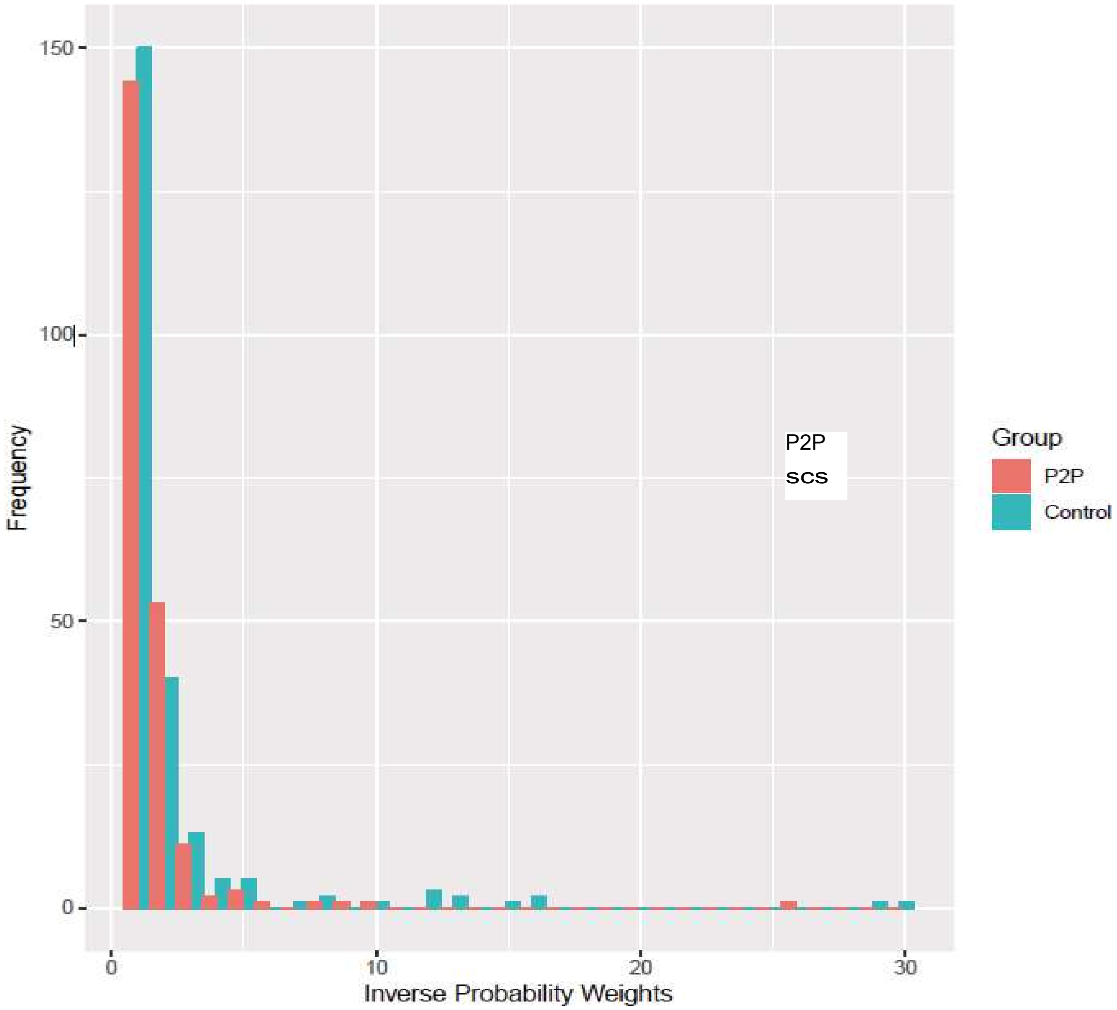

Abbreviations: P2P, peer-to-peer; SCS, standard community services.

Table 3 presents results based on a covariate-adjusted logistic regression model to estimate outcome proportions in each group. The raw estimates are calculated proportions from the raw data and are unadjusted for covariates. Table 3 shows estimated probabilities of outcome that are quite different from the raw estimates. This large discrepancy in percentages between raw and model-based estimates indicates that a covariate-adjusted single regression model is not an appropriate analysis method for these data. Therefore, we adopted a propensity score model for analysis. See "Building a Propensity Score Model" for additional details.

© 2021 Schwei RJ et al. *JAMA Network Open.*

Table 3. Comparison of Raw Estimates and Model Estimates

	Raw estimates, No. (%)		Model estimates, No. %			
	SCS (n = 234)	P2P (n = 222)	SCS (n = 227)	P2P (n = 218)	OR (95% CI)	*P* value
UC	31 {13.2)	13 {5.9)	69.6	41.3	3.26 {1.26-8.45)	.015
ED visit	48 {20.5)	48 {21.6)	22.0	25.7	0.81 {0.42-1.57)	.534
Hospitalizations	48 {20.5)	69 {31.1)	1.4	2.3	0.58 {0.31-1.11)	.100
Primary composite measure	94 {40.2)	101 {45.5)	51.0	56.0	0.82 {0.47-1.43)	.482

Abbreviations: ED, emergency department; OR, odds ratio; P2P, peer-to-peer; SCS, standard community services; UC, urgent care.

Aim 1 and Aim 2 Analysis

For aim 1, we used our propensity score model, taking intracluster correlation within program into account as a random effect to estimate the percentages of any UC visits, ED visits, and hospitalizations in each group. We also used the propensity score model to conduct mixed effects logistic regression models that compared the composite use between the P2P and the SCS groups. For aim 2, we used the propensity score model to conduct a difference of differences analysis, which estimated scores for each measure for each group at baseline, 6 months, and 12 months. Again, this technique accounted for cluster correlation with program as a random effect.

We then used an analysis of variance approach to compare the differences in scores for each measure between baseline and 6 months and between baseline and 12 months for the P2P group vs the SCS group.

We had missing data due to dropout of participants and because some participants died or transitioned to a higher level of care before month 12. As a sensitivity analysis and to account for the dropouts {n = 35 [7.7%]}, we compared the baseline characteristics of the dropouts and the participants who completed the study; those who dropped out were mostly Hispanic women from the Rochester site. We performed the same propensity score analysis of the aim 1 and aim 2 outcomes with and without the dropouts, and we saw no differences in our

© 2021 Schwei RJ et al. *JAMA Network Open.*

findings. Also, as another sensitivity analysis and to account for the competing events of death and transitioning to a higher level of care, we performed the same propensity score analysis, including death and higher level of care, as part of the composite primary outcome; our findings were the same in this sensitivity analysis as in the main analysis.

Sample Size Justification

We were unable to recruit the target number of 720 older adults during the strict study time frame, so we recalculated the sample size needed to detect a 0.27 effect size in the intermediary outcomes of health status and QOL, depressive symptoms, anxiety symptoms, and loneliness. Our total sample size of 456 older adults provided more than 0.80 power to detect a 0.27 effect size in the intermediary outcomes. Because we were unable to randomly assign participants to the study groups, this power analysis is only approximate, as the calculation assumes comparable P2P and SCS groups.

© 2021 Schwei RJ et al. *JAMA Network Open.*
